# Diagnosis, monitoring and prevention of exposure-related non-communicable diseases in the living and working environment: DiMoPEx-project is designed to determine the impacts of environmental exposure on human health

**DOI:** 10.1186/s12995-018-0186-9

**Published:** 2018-02-05

**Authors:** Lygia Therese Budnik, Balazs Adam, Maria Albin, Barbara Banelli, Xaver Baur, Fiorella Belpoggi, Claudia Bolognesi, Karin Broberg, Per Gustavsson, Thomas Göen, Axel Fischer, Dorota Jarosinska, Fabiana Manservisi, Richard O’Kennedy, Johan Øvrevik, Elizabet Paunovic, Beate Ritz, Paul T. J. Scheepers, Vivi Schlünssen, Heidi Schwarzenbach, Per E. Schwarze, Orla Sheils, Torben Sigsgaard, Karel Van Damme, Ludwine Casteleyn

**Affiliations:** 1 Division of Translational Toxicology and Immunology, Institute for Occupational and Maritime Medicine (ZfAM), University Medical Center Hamburg-Eppendorf (UKE), Hamburg, Germany; 2 Faculty of Public Health, Department of Preventive Medicine, University of Debrecen, Debrecen, Hungary; 3 Division of Occupational and Environmental Medicine, University of Lund, Lund, Sweden; 4 Karolinska Institutet, Institute of Environmental Medicine (IMM), Stockholm, Sweden; 5 Tumor Epigenetics Unit, Ospedale Policlinico San Martino, National Cancer Institute, IRCCS and University of Genoa, DISSAL, Genoa, Italy; 6 European Society for Environmental and Occupational Medicine, Berlin, Germany; 7 Cesare Maltoni Cancer Research Center, Ramazzini Institute, Bentivoglio, Bologna, Italy; 8 San Martino-IST Environmental Carcinogenesis Unit, IRCCS, Ospedale Policlinico San Martino, National Cancer Institute, Genoa, Italy; 9 Social and Environmental Medicine, Institute and Outpatient Clinic of Occupational, Friedrich-Alexander-University Erlangen-Nurnberg, Erlangen, Germany; 10 Institute of Occupational Medicine, Charité Universitäts Medizin, Berlin, Germany; 11 WHO European Centre for Environment and Health, Bonn, Germany; 12 Biomedical Diagnostics Institute, Dublin City University, Dublin, Ireland; 13 Norwegian Institute of Public Health, Oslo, Norway; 14 Center for Occupational and Environmental Health, Fielding School of Public Health (FSPH), University of California Los Angeles (UCLA), Los Angeles, USA; 15 Radboud Institute for Health Sciences, Radboudumc (Radboud university medical center), Nijmegen, the Netherlands; 16 National Research Center for the Working Environment, Copenhagen, Denmark; 17 Department of Public Health, Section Environment, Occupation & Health & Danish Ramazzini Centre Aarhus, Aarhus University, Aarhus, Denmark; 18 Department of Tumor Biology, University Medical Center Hamburg-Eppendorf (UKE), Hamburg, Germany; 19 Department of Histopathology, Central Pathology Laboratory, St James’s Hospital, Trinity translational Medicine Institute, Dublin, Ireland; 20 Center for Human Genetics, University of Leuven, Leuven, Belgium

**Keywords:** Noncommunicable diseases, Human biomonitoring, Environmental/occupational exposure to xenobiotics

## Abstract

The WHO has ranked environmental hazardous exposures in the living and working environment among the top risk factors for chronic disease mortality. Worldwide, about 40 million people die each year from noncommunicable diseases (NCDs) including cancer, diabetes, and chronic cardiovascular, neurological and lung diseases. The exposure to ambient pollution in the living and working environment is exacerbated by individual susceptibilities and lifestyle-driven factors to produce complex and complicated NCD etiologies.

Research addressing the links between environmental exposure and disease prevalence is key for prevention of the pandemic increase in NCD morbidity and mortality. However, the long latency, the chronic course of some diseases and the necessity to address cumulative exposures over very long periods does mean that it is often difficult to identify causal environmental exposures.

EU-funded COST Action DiMoPEx is developing new concepts for a better understanding of health-environment (including gene-environment) interactions in the etiology of NCDs. The overarching idea is to teach and train scientists and physicians to learn how to include efficient and valid exposure assessments in their research and in their clinical practice in current and future cooperative projects.

DiMoPEx partners have identified some of the emerging research needs, which include the lack of evidence-based exposure data and the need for human-equivalent animal models mirroring human lifespan and low-dose cumulative exposures. Utilizing an interdisciplinary approach incorporating seven working groups, DiMoPEx will focus on aspects of air pollution with particulate matter including dust and fibers and on exposure to low doses of solvents and sensitizing agents. Biomarkers of early exposure and their associated effects as indicators of disease-derived information will be tested and standardized within individual projects. Risks arising from some NCDs, like pneumoconioses, cancers and allergies, are predictable and preventable. Consequently, preventative action could lead to decreasing disease morbidity and mortality for many of the NCDs that are of major public concern. DiMoPEx plans to catalyze and stimulate interaction of scientists with policy-makers in attacking these exposure-related diseases.

**Electronic supplementary material:**

The online version of this article (10.1186/s12995-018-0186-9) contains supplementary material, which is available to authorized users.

## Background

Adverse health outcomes because of exposure received in the living and working environments in combination with lifestyle have been estimated to be responsible for up to 75% of global noncommunicable diseases (NCDs) [1, 2]. Chronic diseases resulting from these exposures provide a major contribution not only to the NCD burden but also to the resulting increase in health costs. Since most of these diseases are preventable, appropriate health policies should concentrate on this major societal challenge.

In 2010, about 40 million people died worldwide from NCDs, including cancer, diabetes, and chronic cardiovascular, neurological and lung diseases [3]. This represents an increase from 60% of total deaths attributed to these diseases in the year 2000 to 70% (total deaths) within 10 years (see Additional file 1: Info Box 1, for more details). In 2015, the World Health Organization (WHO) ranked environmental exposures among the top risk factors for chronic disease mortality [4]. Pollution (from air, soil, water) is one of the leading causes of death from NCDs (for other environmental factors see Table 1). Worldwide, diseases related to environmental pollution were responsible for 9 million premature deaths in 2015 - three times as many deaths as from AIDS, tuberculosis and malaria combined [5]. Every year, environmental risks – such as indoor and outdoor air pollution, second-hand smoke, unsafe water, lack of sanitation and inadequate hygiene – take the lives of 1.7 million children under 5 years, reported WHO in 2017 [6]. Ambient air pollution alone is estimated to cause 7 million premature deaths per year (recently highlighted in the Global Burden of Disease (GBD project [7]). Data from the GBD study group demonstrate a strong link between both indoor and outdoor air pollution exposure and cardiovascular disease (CVD), as well as between air pollution and cancer [8]. In some parts of the European Union (EU), air pollution causes a reduction in the average life expectancy of more than one year [9, 10].

**Table 1:** Synopsis

The main purpose of the European Cooperation in Science and Technology program is to provide a framework for international cooperation among researchers and other professionals. By bringing together experts in significant areas of human life and development, opens up the possibilities of new ideas, approaches and solutions. The European Cooperation in Science and Technology COST program is founded partially by the member states, who delegate the management committee members. The Action Diagnosis, Monitoring and Prevention of Exposure-related Noncommunicable Diseases (DiMoPEx) fosters capacity-building by bringing together basic scientists, clinical researchers and practitioners in the relevant (sub-)disciplines and organizing interdisciplinary collaboration and training in research that addresses the societal challenges outlined above. Members aim to implement new concepts in joint interdisciplinary research and training initiatives to enhance networking between expert centers and offer a platform for interdisciplinary collaboration between researchers across Europe. DiMoPEx also aims to attract and focus the interests of the next generation of early career investigators on key emerging issues of exposure-related disease burden and various aspects of exposure assessment sciences.
The predominant goal is to help scientists, physicians and health officials to prevent and reduce health impacts associated with various exposure scenarios and train highly skilled researchers of health-environment (including gene-environment) interactions in the etiology of exposure- related NCDs within seven working groups
The overarching idea of the DiMoPEx project (http://dimopex.eu/working) groups is to teach and train about how to learn to include evidence-based exposure assessment (in research and clinical settings). Using modern methods such as ambient monitoring and human biomonitoring methods (WG1, WG 2), the various biomarkers of effect and susceptibility alongside with the clinical diagnostic methods and biomarker-based evaluation of lifestyle factors (WG3, WG 6) can be combined, resulting in the development of cooperative projects that are too broad for coverage by individual disciplines (i.e. epidemiology or traditional environmental medicine). Within several joint research projects, DiMoPEx partners are already focusing on the impact of pollution on human health. The projects are concentrating on several pollutants (particulate mass fractions PM2.5 and PM10, a range of metals, inorganic gases and organic compounds) in living and working environments and their health impacts [138].
The DiMoPEx Action anticipates initiating health research with important benefits for public health and the healthcare system of the European Community. DiMoPEx will catalyze and stimulate interaction of scientists with policy-makers on exposure-related diseases of concern to society (see below, WG 7 for more details on cooperation with the WHO scientists, implementation of the new knowledge, involving external partners and policy makers). See below for detailed working groups description.

The concept of the “exposome” as the total of all external exposures, along with individual susceptibility due to genetic, age-related, and other vulnerabilities, is gaining increasing credence from both the scientific and clinical communities [11, 12]. Pollutants, food additives, chemicals found in cosmetic products and therapeutic exposure (chemo−/ radio therapy) are prime examples of such cumulative exposures. Certain pesticides, such as organophosphates, are examples of man-made chemicals to which large populations in agricultural communities are exposed, as well as consumers via their diet, and contribute to neurotoxicity in human populations worldwide [13–15]. The compromising of health (effect measure modifications, EMM) is possible through lifestyle factors such as smoking, alcohol abuse and bad nutrition/obesity, as well as through interactions between these. For example, smoking increases the risk of lung cancer (through co-exposure to asbestos, radon or arsenic) from < 20% (exposure alone) to over 80% excess risk because of the synergistic effects [16]. Health hazards also arise from the globalization of trade [17] and production processes with direct and indirect environmental and occupational health impacts [18, 19]. Further, new hazards are continuously being discovered, such as those related to the introduction of nanoproducts in industrial and consumer goods [20].

The long latency periods, combined cumulative exposures and chronic course of diseases often makes it difficult to identify environmental/occupational exposure as the cause of NCDs [21, 22]. One source of exposure may cause several outcomes and also different types of exposure may affect the same disease outcome; for example, air pollution has been linked to a number of common diseases, including cardiovascular, cerebrovascular, respiratory, reproductive, neuro-developmental and neuro-degenerative diseases. [23] Conversely, multiple exposures may have a cumulative effect on the same target organ.

At the patho-physiological level, exposure-related NCDs arise as a result of interactions between internal (genetic, epigenetic, hormonal, aging etc.) factors and external (occupational/environmental) influences [24]. In recent years, enormous progress in the exploration of genetic and epigenetic factors and resulting disease risks has been made. This knowledge has already found its way into the contents of academic teaching programs in medical schools and postgraduate courses (e.g. in molecular epidemiology, neurosciences, personalized medicine). In contrast, the other major and modifiable dimension of pathogenesis, the influence of occupational/environmental exposure and lifestyle factors, has received comparatively little attention. Current figures published by WHO (see Additional file 1: Info Box 1) indicate an urgent need for an update in the research and training potential concerning environmental health issues, and in implementing public health research across Europe, with an interdisciplinary evidence-based orientation in the natural sciences, public health and medicine.

### Outline

This review assesses the current status and future needs of the multicenter European COST Action DiMoPEx (http://www.cost.eu/COST_Actions/ca/CA15129). The separate sections represent the identified current research objectives and future goals of the DiMoPEx action. It reflects the structure of this multicenter action with 7 working groups (http://dimopex.eu/working), highlighting the role of individual working groups within the DiMoPEx framework and the specific methods provided by individuals groups for the ongoing and planned collaborative projects. A short description of the ongoing interdisciplinary research projects is also provided demonstrating how the evidence-based exposure data can be applied for the diagnosis and monitoring of exposure-related NCDs (from the perspective of the action partner).

## DiMoPEx project goals identified by the project partners

### How to improve diagnosis, monitoring and prevention of NCDs?

#### Current status and future needs to be addressed

DiMoPEx partners recognize an important research need: to link the living and working environment with disease prevalence in order to prevent the pandemic increase in NCD morbidity and mortality. Public health benefits may range from effective preventative measures to early detection of possible adverse health outcomes. Four of the currently identified emerging research tasks pursued by DiMoPEx include the following:

1. **To face the difficulties in NCD diagnosis and monitoring of disease progress**
  Many ongoing long-term studies focusing on early signs of related chronic diseases account insufficiently for environmental/occupational determinants of health. Other studies addressing health outcomes in relation to exposures in the living and working environment do not sufficiently account for existing knowledge regarding appropriate exposure measures in their study designs (i.e. some record ever/never occupational exposure or self-reporting of specific chemicals, leading to exposure misclassification and biased results). The effects of multiple exposures and EMM within the same target organ should also be addressed. It is time now to take a closer look at the living and working environment and focus on evidence-based exposure data that has the potential to correlate exposure with disease, which otherwise provides an obstacle to evidence-based recommendations for primary and secondary NCD prevention.
2. **To focus on biomarkers of early response and appropriate human-equivalent animal models (carcinogenicity bioassays to provide a basis for evidence- based interventions**
  Evidence-based interventions have already successfully limited exposure to many known and probable carcinogens, including tobacco, arsenic, asbestos, benzene, vinyl chloride and air pollution. However, among NCDs, cancer is still the second leading cause of death: in 2014 about 591,699 of people died from cancer in the United States alone [25]. Cancer is an extremely complex disease, not easy to control, and one about which there is insufficient knowledge in terms of etiology. To provide a solid scientific basis for cancer prevention, it is necessary to increase our knowledge about cancer etiology. Basic as well as preventative and clinical research should be developed. In this research, well-designed experimental animal studies [26] and biomarkers of early response should play a central role (carcinogenicity bioassays).
3. **To focus on air pollution as one of the major factors responsible for NCD mortality**
  There is a strong link between both indoor and outdoor air pollution exposure and CVD, as well as between air pollution and cancer. Knowledge of what it is that makes a particle toxic may provide better exposure metrics in epidemiology studies, lead to more efficient abatement strategies to reduce emissions of the most hazardous air pollutants and allow for production of nanoparticles that can be shown to be benign. Being able to predict the toxicity of particulates based on knowledge of size, composition and material properties would also be a prerequisite for reducing the need for extensive toxicity testing of new nano-materials. The oxidative potential of particles is considered by many to be a promising metric to predict particle toxicity.
4. **To recognize the need for the public-health protection through cooperation with policy-makers**
  To benefit societies and enhance the wellbeing of populations and decrease morbidity and mortality from exposure-related NCDs, there is a need for innovation in public health and environment policy and in the business practices of certain industries, leading to healthier environments, as well as a better understanding of risk communication, including its ethical aspects. There is a need to catalyze and stimulate interaction between scientists and policy-makers in respect of exposure-related diseases of concern to society. The predominant goal should be to help scientists, physicians and health officials to prevent and reduce health impacts associated with various exposure scenarios and to train highly skilled researchers for the future labor market.

## Implementation of the research goals within the framework of the 7 WGs

The identification of a xenobiotic chemical and the documentation of the degree and extent of exposure by the WG 1 project is fundamental to the investigation of the disruptive effects of that exposure and its consequences for NCDs, which are the specialist interests of the WG 5, WG 2 and WG 6 projects in determining the biohazard consequences in carcinogenicity, genotoxicity and health effects. WG 3, WG 4 and WG 7 support other groups with knowledge on epidemiology and/or risk communication and canvassing meetings with policy makers to influence environmental/occupational laws, funding groups, etc.

Detailed descriptions of methods applied, issues to be concentrated on within the project and further examples of current activities are summarized in the following sections.

## WG 1 advancing towards evidence-based exposure data

### Exposure assessment – From environmental to individual exposure

**Fig. 1:**
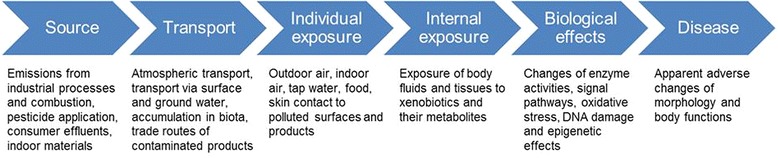
The wide spectrum of sources needed to ensure accurate exposure assessment

An accurate exposure assessment needs consideration of a wide spectrum of sources, the different pathways and routes of exposure, and the environmental and physiological effects of the xenobiotics [27] (Fig. 1).

The most prominent sources of xenobiotic chemical exposure are emissions from industrial processes and engine exhausts, emissions from other combustion processes, residues from pesticide and biocide applications, emissions from consumer populations via waste effluents (solid waste or wastewater), and indoor air emissions from building materials, consumer products and furniture. People are mainly exposed to this pollution via outdoor and indoor air, tap water and contaminated food, as well as direct skin contact with contaminated surfaces and dermally-applied products, such as cosmetics and personal care products [28–33]. In some special cases, exposure may also occur during rainfall, from surface water, dermal contact or oral ingestion of contaminated soil, from applications of pesticides, biocides and other chemical products [34]. Moreover, lifestyle behavior such as. smoking and the consumption of functional food, nutriceuticals and the application of pharmaceuticals, exacerbate the broad spectrum of chemical exposure experienced by an individual. The manifold emission sources, polluted materials and exposure scenarios determine that extraneous chemicals gain access to the body using all possible routes, most especially by inhalation, oral ingestion and via the dermis.

The assessment of chemical exposure has two main aims. Firstly, the individual pollution agents have to be clearly identified. Secondly, qualitative detection also requires ascertainment of the hazards inherent in these xenobiotics. For risk assessment, however, both the qualitative character of exposure and the extent of the exposure have to be estimated. Some approaches of exposure assessment already enable the attainment of both of these goals, e.g. by a non-target procedure which may also enable a semi-quantitative determination of the analytes. However, for those approaches in which specific metabolites have to be assessed, the prior identification of the chemical agent is indispensable before targeted measurement can be implemented.

**Fig. 2:**
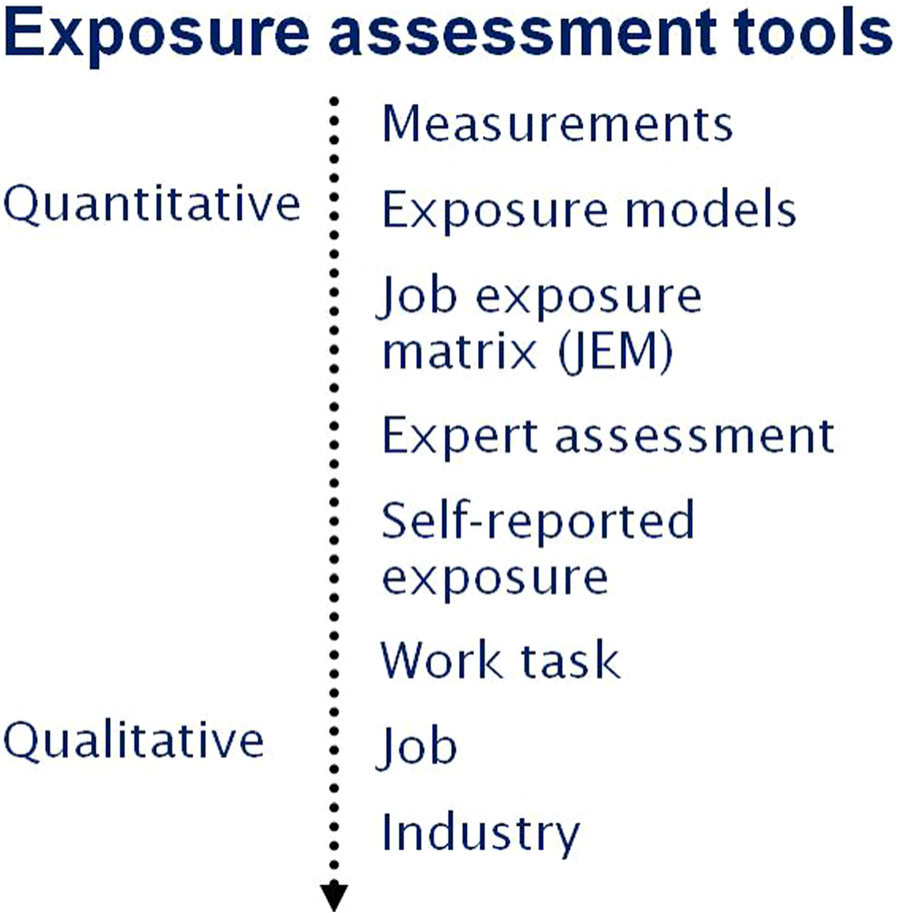
Quantitative estimation of the exposure performed by a direct or indirect approach; example from the occupational medicine

A quantitative estimation of exposure can be performed by a direct or an indirect approach (see Fig. 2). A direct monitoring approach requires determination of the extent of exposure of an individual to a chemical by assessment either externally, internally or as metabolized products. External measurements of ambient exposure can be made from air contamination by that chemical or the contamination of the skin. The internal exposure to a chemical suffered by an individual is by conventional human biological monitoring and the measurement of the unmodified agent, as well as its metabolites and reaction products in blood and urine (see also the later section on human biological monitoring (WG2)). Inevitably, the levels of internal exposure are most strongly connected with the effective dose and the subsequent toxic effects [35]. To use data from individual ambient exposure for risk assessment effectively then it is necessary for an additional calculation about the absorption efficiency to be made, e.g. by using minute volume, respiratory retention or dermal absorption rate. Indirect approaches of exposure assessment (i.e. dispersion models or other exposure models, questionnaires on exposure scenarios, and questionnaires on food intake or exposure situations) can also be taken as a basis for estimating the levels of environmental contamination. Data from indirect approaches have to be extrapolated to the effective dose in the population by considering pollutant transport processes, accumulation and fate processes in the environment, exposure scenarios, demographic and geographic attributes, lifestyle behavior, human constitution and the pharmacokinetics of the agent. Moreover, an estimation of individual exposure has to include the intra-individual variability of these extrapolation factors within the population [36, 37]. Each extrapolation model should be validated in respect of its performance and uncertainty. Regardless of which approach was used for the assessment of the recent extent of exposure, these might be important for a reasonable risk assessment and for contemplation of the duration of exposure [38].

The tasks of the WG1 “Exposure assessment” project encompass the analysis of skills, expertise and capacities regarding exposure assessment within the consortium, the dissemination of resources and information on assessment procedures and quality assurance, as well as the development and expansion of capabilities and capacities. The most important tasks are the identification of limitations or crucial gaps in knowledge about exposure quantitization and exposure-effect associations, as well as preparing effective solutions for closure of these knowledge gaps. In particular, the WG 1 and WG 2 project groups are providing a sustainable research and training program in the field of exposure science (human biomonitoring, ambient monitoring) for the other DiMoPEx partners.

## WG 5 hazards characterization, risk identification: Carcinogenicity bioassays

### Diagnosis of cancer as NCD needs biomarker(s) of early effect (detection of preclinical lesions) and new animal study approaches

There is a need for human-equivalent animal models mirroring human lifespan and low dose cumulative exposures. The laboratory rat has served as the traditional animal model of choice for research and regulatory developmental and reproductive toxicity testing conducted to support human health hazard identification and risk assessment. The laboratory rat has been more thoroughly characterized than have other species in these research fields, especially when identifying likely human carcinogens. However, with new insights into toxicology, novel integrated experimental approaches for hazard identification are needed with human-equivalent animal models in rodent bioassays for primary prevention (see Additional file 1: Table S1; [39, 40] and Info Box 2) for more information on animal models and Organisation for Economic Co-operation and Development (OECD) guidelines [41].

When conducting cancer bioassays, it is important to investigate the effect of low doses and a systematic dose-calibration study should be performed in an appropriate rodent model in order to identify the relevant administered oral dose of the test substance that results in biomarker concentrations (e.g. urine, serum) comparable to those observed in a human population [42]. Cancer is a complex disease with diverse etiology; see examples of exposure related cancer in Additional file 1: Info Box 3, Table S2 in the supplementary). The neoplastic response depends not only on the kind of agent, its physicochemical and toxicological properties, the mode of exposure, and the type of animal but also, to a great extent**,** on the latency of the tumor, which varies widely and may be very long. Experimental findings indicate that the latent neoplastic potential for causing a tumor increases with the length of the observation time or age. Thus, experimental carcinogenicity trials should continue until spontaneous animal death and not be cut short. To give a clearer explanation, one of the the DiMoPEx partners compared, in preliminary research, human deaths from malignant tumors at the Hospital of Trieste, in 1989, with rat deaths from malignant tumors in the RI animal facility belonging to control groups, in 1984–1994. Figure 4, which refers to the cumulative prevalence of animals and humans with malignant tumors, histopathologically observed by age at death, shows that 80% of tumors arise after the age of 65 years in humans, which corresponds to 104 weeks in rats [43]. According to the OECD, rats should be sacrificed at 112 weeks of age at death [43], which corresponded to 104 weeks after the start of the treatment. If these animals had been sacrificed at 112 weeks of age (comparable to 65 + age in humans) then the majority of tumors would have been missed. At the Cesare Maltoni Cancer Research Center (CMCRC), studies have been conducted on more than 200 compounds present in the industrial and the general environment, including vinyl chloride, benzene, formaldehyde, trichlorethylene, fuels and their components and additives, pesticides, and recently aspartame, the most widely diffused artificial sweetener in the world. The results from the CMCRC studies have provided the scientific basis for lowering exposure levels to various agents present in places of work and in daily life. They have also formed the basis for rules and regulations of primary prevention, even if sometimes many years have passed before confirmation of their carcinogenicity in humans (see Additional file 1: Table S3).

#### Current two-year experimental schemes may mask a carcinogenic response

**Fig. 3:**
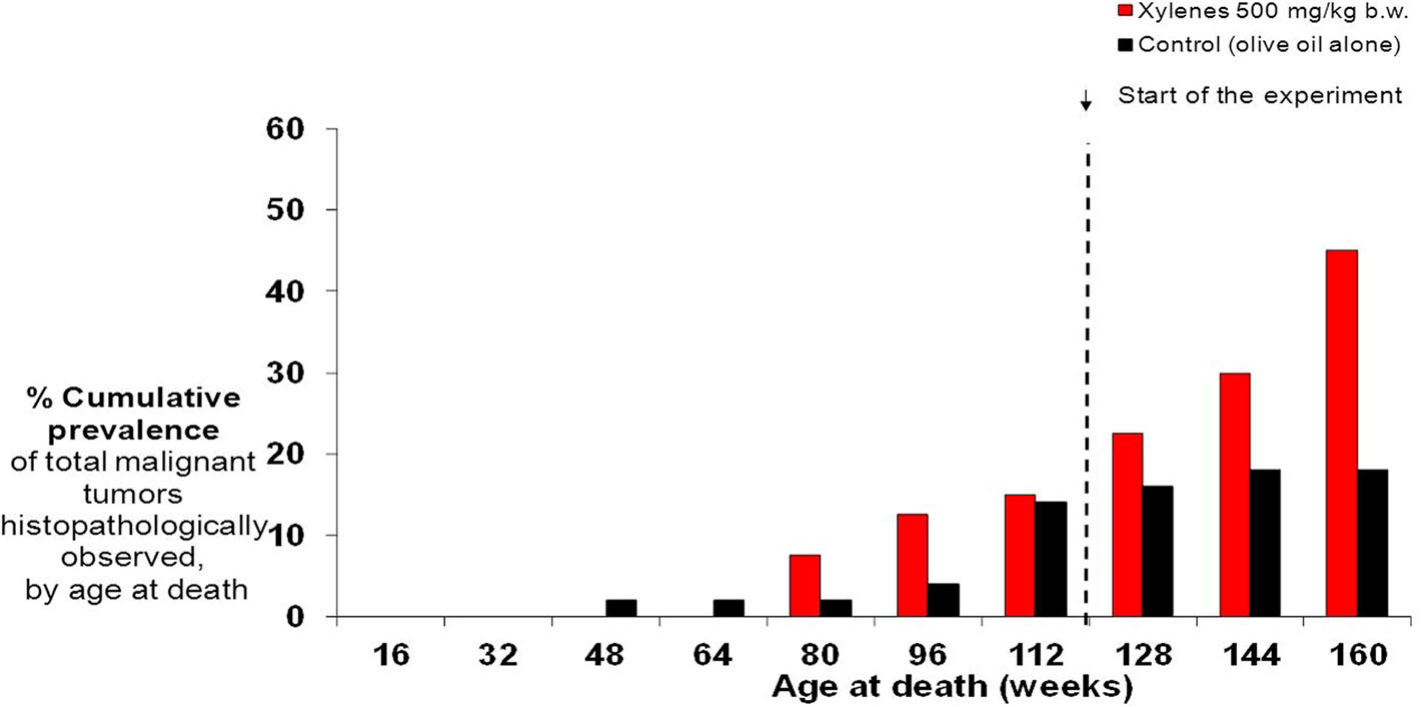
New study protocols needed for animal models. Animal models for carcinogenicity bioassays: Hazard identification: carcinogenic effects may be observed later than 112 weeks after xylene exposure

Cutting short an experiment after two years may mask a possible carcinogenic response, as in the following cases with xylene and mancozeb (see Additional file 1: Table S4) The increase in total malignant tumors, oral cavity carcinomas and hemolymphoreticular neoplasias was only observed for xylene administration after 112 weeks of age (Fig. 3). It should be noted that during exposure tests for xylene, performed by the United States National Toxicology Program, the rats were sacrificed after 104 weeks of treatment without any carcinogenic effect being found [44, 45]. With mancozeb administration, a strong increase in malignant tumors of the thyroid gland in males and female rats was also observed after 112 weeks of age [46]. In demanding that chronic animal studies be terminated after 2 years, regulatory agencies may lose information that is important for extrapolation of the data from animals to humans, most especially for chronic diseases with a long latency time.

#### An integrated experimental approach

To satisfy the need to consider multiple effects (e.g., cancer and non-cancer) across multiple life stages and to reduce the overall number of animals required for separate studies of these end-points, the adaptation of the carcinogenicity bioassay to integrate additional protocols for comprehensive long-term toxicity assessment was recently proposed. The central aim of the methodology proposed in the integrated experimental approach was to maximize the breadth of outcomes assessed and to increase the sensitivity of testing beyond that in commonly used protocols. This should yield more reliable and inclusive information on many important end-points. In this experimental design, rats from the same generation are used for studying chronic toxicity and carcinogenicity outcomes and distributed into parallel satellite experiments for detecting reproductive/developmental toxicity, thus minimizing variables between different arms of the multi- end-point investigation [47]. This protocol is a incentivizing proposal to regulatory scientists and the scientific community in general. By conducting such integrated bioassays, scientific evidence of risk assessment would be enhanced and expanded, by gathering sufficient and rapid information about several adverse effects in a unique protocol for protecting public health.

### Biomarker of early response to assess the effects of preventive measures and identify individuals at high risk of developing a particular NCD

Efficient patient management relies on early diagnosis of diseases and monitoring of disease progression. In this respect, significant efforts have been made to find informative, blood-based biomarkers or liquid biopsy. As the individual genomic, epigenomic and transcriptomic profiles of diseases become more and more elucidated, the applicability of circulating nucleic acids and exosomes have the potential to complement the existing blood biomarkers in future. In addition, the blood-based detection of disease-specific genetic aberrations, such as mutations, microsatellite alterations and epigenetic modulations in circulating free DNA (cfDNA), or quantitative changes in cfDNA, RNA, microRNAs (miRNAs) and exosomes, represent highly promising approaches for the risk assessment of various diseases. Investigations of these molecular alterations have also revealed an impact on gene expression, resulting in aberrant regulation of disease-specific signal transduction pathways. For the most acute clinical syndromes, it is likely that multiple markers rather than a single marker will give the best diagnosis and prognosis.

The use of the cytokinesis-block micronucleus (CBMN) cytome assay could also be relevant for clinical and epidemiological studies and for preventative interventions, because it could allow the identification of individuals at high risk of developing a given disease and could even qualify as an intermediate biomarker to assess the effects of preventative measures. In prospective studies evaluating large cohorts of disease-free subjects, an increase in micronucleus frequency (MN) in peripheral blood lymphocytes was associated with an increased cancer risk at the population level, providing suggestive evidence that this biomarker may be predictive of cancer risk [48]. Increased MN frequency was also detected in peripheral lymphocytes of subjects affected by cancer-associated congenital syndromes characterized by deficiencies in the DNA damage response [49]. Many studies also showed an increased MN frequency in peripheral lymphocytes in untreated patients with cancer or pre-neoplastic lesions [50], neurodegenerative diseases [51], CDV and diabetes [52].

### Potential biological effect markers – Circulating nucleic acids in human blood

Circulating nucleic acids are promising blood-based biomarkers because of their informative and disease-specific features. Their deregulated levels are associated with tumor genesis, tumor progression, metastases and drug resistance in cancer patients, and reflect physiological and pathological processes of different diseases. Circulating nucleic acids (in plasma or serum) may serve as a “liquid biopsy” that is useful for numerous diagnostic and prognostic applications of different (malignant and benign) diseases, while avoiding tissue biopsies by invasive methods. This minimally invasive procedure allows the repeated taking of blood samples, providing the ability to follow quantitative measurements and genetic or epigenetic changes during the natural course of the disease, facilitating treatment decisions [53, 54].

**Fig. 4:**
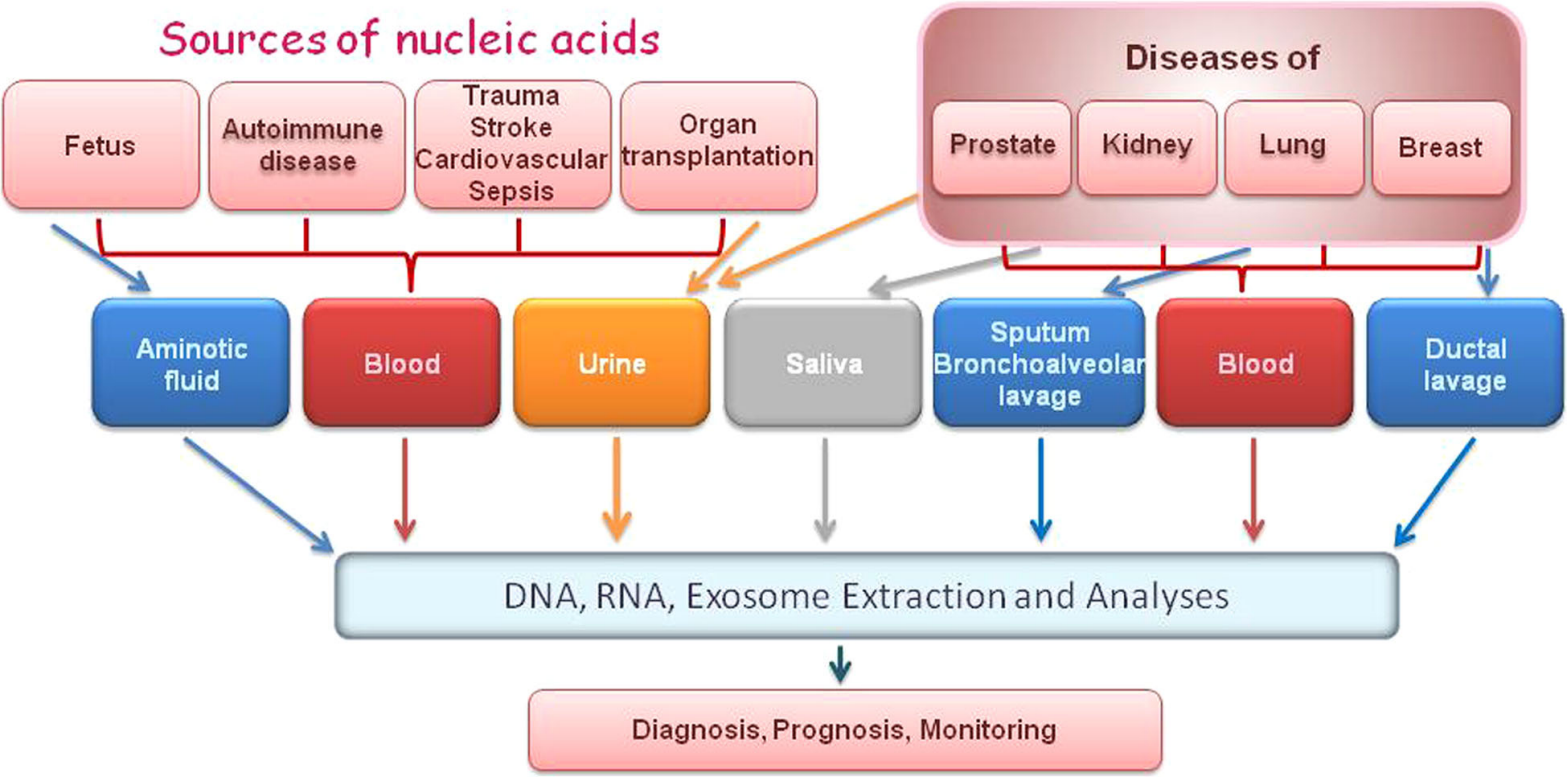
Sources of nucleic acids and NCDs

Nucleic acids are usually released (Fig. 4) during cellular stress or tissue injury into the blood circulation. Their release is associated with inflammatory responses caused by a coordinated expression of numerous genes that initiate, sustain and propagate immune responses and tissue remodelling [55]. This increased cell turnover and impaired blood clearance are possible reasons for the elevated or deregulated levels of circulating nucleic acids in critical disease conditions where organs responsible for elimination of by-products are damaged (as a consequence of systemic inflammation). The release of nucleic acids into the blood circulation occurs during the processes of apoptosis and necrosis. While apoptotic cell death leads to controlled inter-nucleosomal cleavage of genomic DNA, necrotic cell death leads to a discharge of large genomic DNA fragments [56]. Apart from their passive release during cell death, nucleic acids can also be actively excreted into the blood circulation by microvesicles, such as exosomes. Exosomes and their cargo are thought to play an important role in cell-to-cell communication by influencing the recipient cell phenotype [57].

The concentrations of cfDNA are elevated (as an early signal) in the blood circulation after onset of disease and reach the highest level in patients with disease-specific complications and a high mortality risk. Accordingly, the elevation in cfDNA levels is not specific for a specific disease and varies among patients within a patient cohort, but may correlate with the severity of disease. Since cfDNA levels may change during the course of disease and in parallel with the severity of disorder, they could provide a useful marker for the assessment of adverse outcome, allowing clinicians to make a rapid risk stratification for more rational therapeutic decisions [58].

Apart from cfDNA, much attention and effort have been put into the study of cell-free microRNAs (miRNAs) (see Additional file 1: Table S5, [59–61]). The expression of these small, non-coding RNA molecules is often tissue-specific and in many pathological conditions characteristically deregulated. The clinical relevance of cell-free miRNAs [59] as diagnostic and prognostic markers has been documented for a variety of diseases [60]. Besides, miRNAs that are actively released in exosomes into the blood circulation, can be transferred to recipient cells [61] and can be functional there by respressing their mRNA targets [62]. Thus, exosomes may serve as suppliers of disease-derived genetic information and, consequently, transform their host cell as well. Moreover, exosomal miRNAs stimulate cellular signalling and regulate metabolic functions and homeostasis. The amounts of secreted exosomes as well as their contents of miRNAs have been associated with tumor development and progression, cell migration and proliferation of tumor cells leading to metastasis [63] . (see Additional file 1: Table S5).

### Epigenetic markers in early detection of NCDs

Unlike genetic alterations, which can be stably acquired during the life-course, epigenetic modifications (see Additional file 1: Table S6) are dynamic, tissue-specific and can be characteristic of a disease. In this respect, epigenetic alterations may be utilized as biomarkers of exposure and disease and serve as biomolecular sensors for preventive surveillance [64].

Environmental and occupational factors induce epigenetic alterations that can contribute to the onset of NCDs, of which cancer is one of the most prevalent. Occupational exposure to chemicals (e.g. benzene), dusts (e.g. from manufacturing of leather or woods) and/or industrial processes (welding, metallurgy) can be related to cancer and the carcinogenic process is linked to changes in DNA methylation, particularly in its early phases. Recently, the epigenetic involvement and contribution of 12 chemicals and associated occupations were evaluated from the literature by the International Agency for Research on Cancer (IARC). Human carcinogens related to environmental occupational hazards classified as Group 1 were considered, specifically the three carcinogens aflatoxin, benzene and benzo[a]pyrene), where several studies have reported an epigenetic effect [65]. Increasing scientific evidence has linked diseases other than cancer with epigenetic alterations and exposure to toxic substances. As an example of trans-generational effects, exposure to toxicants during fetal life could be correlated with neuro-developmental disorders, and epigenetics was considered to be the probable functional phenotype that communicated these diseases [66].

Occupational exposure to specific industrial processes, such as the production and use of nanotubes or fullerenes, can induce epigenetic alterations directly or indirectly through reactive oxygen species (ROS) [67–69]. Occupational asthma [70] and some metabolic diseases can modify the epigenetic status and can contribute to modification of the epigenome. Some neurological diseases, such as Alzheimer’s [71] and Parkinson’s disease [72], have been linked with occupational exposure to organophosphates and to alteration of the DNA methylation landscape in exposed subjects, underlining a possible cause-effect relationship that needs to be further explored.

### Enhancement of genotoxicity and susceptibility markers

#### Human MN

**Fig. 5:**
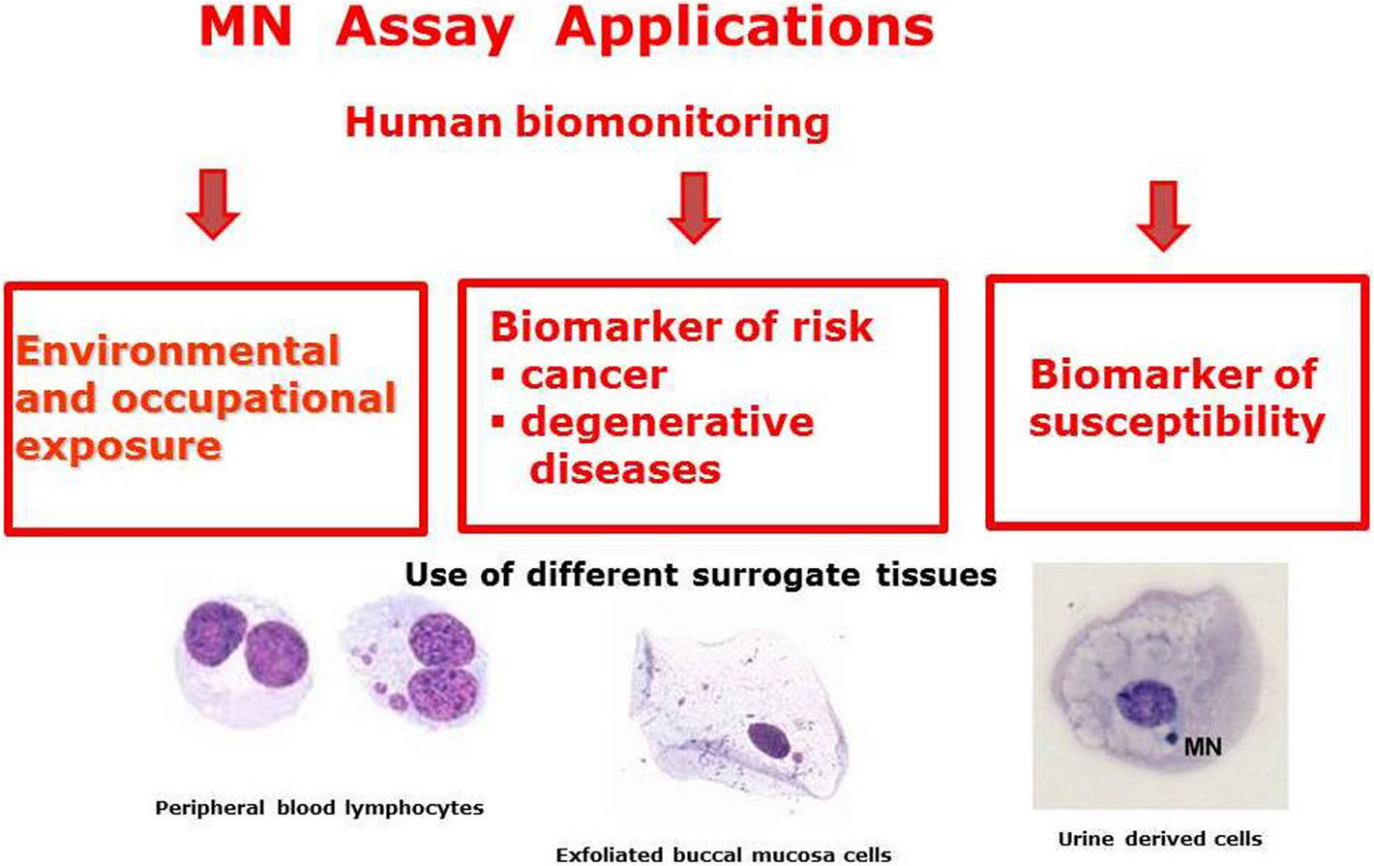
Application of MN assay in human biomonitoring (effect monitoring) after environmental and occupational exposures

The MN test is a measure of the increase in micronucleus frequency in cells and is one of the most successful assays in genetic toxicology because of its ability to detect both structural and numerical chromosomal aberrations [73]. It is one of the most widely applied methods for biomonitoring human populations for evaluating exposure to genotoxic agents and genetic instability. The test was established in different surrogate tissues: peripheral blood lymphocytes and erythrocytes, buccal-exfoliated cells and urine-derived cells (Fig. 5). However, the CBMN cytome assay in peripheral blood lymphocytes is the most validated of the methods.

The MN assay, applied in vitro with different established cell lines and in human cultured peripheral lymphocytes, is recommended as part of the basic battery of tests to screen new chemical agents for genotoxicity, allowing the detection of both clastogenic and aneugenic compounds. Indeed, the OECD has published guidelines for the testing of chemicals using the in vitro and in vivo MN assays [74, 75].

#### Biomonitoring human exposure to genotoxic agents –- CBMN cytome assay in peripheral blood lymphocytes

The CBMN assay is a standard biodosimetry method endorsed by the International Atomic Energy Agency and WHO for measuring exposure to ionizing radiation [76]. The use of the MN test in other exposure scenarios needs to be considered case by case on the basis of the mechanisms of action of the genotoxic agents.

The CBMN assay was largely applied in human populations to evaluate occupational and environmental exposure to genotoxic agents belonging to different chemical classes, with more than 500 associated papers available in the scientific literature. The most frequently investigated groups are hospital personnel, followed by workers in the chemical industry and agricultural workers. The use of the lymphocyte CBMN assay in different exposure scenarios was recently evaluated in the framework of the International Collaborative Project on Micronucleus Frequency in Human Populations (Human Micronucleus project, HUMN) using the systematic review approach [77].

All of the different exposures considered in this review were associated with increased MN frequencies: the average value calculated was 2.5-fold over the background, although the heterogeneity of the available studies and the relevant differences in the quality of the studies do not allow clear conclusions to be drawn. The most pronounced effects, evaluated as increases with respect to the control values, were detected in individuals exposed to metals such as arsenic (6.5-fold), lead (3.8-fold), and chromium (3.5-fold) [78].

Overall, many of the evaluated studies had limitations in study design, recruitment strategy to enrol exposed and control subjects, low statistical power and/or lack of reliable exposure data. The subject selection in many cases did not consider the different tasks in which the workers were involved, the use of protective devices and the known confounding factors for exposed-control matching. Further analyses are needed to elaborate guidelines for the use of the CBMN assay in biomonitoring studies.

#### MN assay in buccal-exfoliated cells

The MN) cytome assay in uncultured buccal-exfoliated epithelial cells is a minimally invasive approach for evaluating genomic damage and cell death in the human aero-digestive tract (for more information on MN assay in buccal-exfoliated cells, see Additional file 1: Info Box 4) [79–83]. Our recent meta-analysis provides evidence for the utility of the MN assay using buccal-exfoliated cells in the pre-screening as well as in the follow- up of pre-cancerous oral lesions. A significant excess of MN in patients compared with matched controls was observed for patient subgroups with oral and neck cancer (meta-mean ratio (MR) of 2.40, 95% CI: 2.02–2.85) or leukoplakia (meta-mean ratio MR 1.88, 95% CI: 1.51–2.35) [84].

The overall objective of the WG 5 project group is to provide research and training programs covering hazard characterization, risk identification and various early effects biomarkers, including carcinogenicity bioassays. The leader of the WG 5 group are acknowledged expert in the field and provide valuable support for the interdisciplinary projects where needed.

## WG 2 human biological monitoring – More than (just) analysis of biomarkers

### Exposures to chemicals and particles

A generic view that can be applied to most uses of biomarkers is their contribution to an understanding of the causal link between environmental exposure(s) and the onset and morbidity of disease. From the perspective of epidemiology, the gaps between cause and health outcome may be bridged by the use of biomarker-based research (WG 2). In occupational and environmental health, the use of biomarkers is embedded in a process termed “human biological monitoring” and defined as *“*the standardized and repeated systematic collection, pretreatment, storage and analysis of body tissues to assess the internal dose of a xenobiotic substance by analysis of the parent substance and/or a product of biotransformation” [85]*.* A much wider definition of biomonitoring also includes biomarkers that do not carry chemical structure information (a “unique signature”) that enables the researcher to link a biomarker value to a specific external factor.

**Fig. 6:**
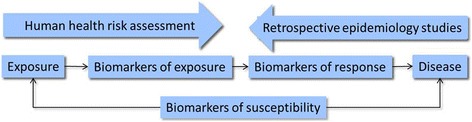
Human biomonitoring: how a specific biomarker can serve as specific aim in a study design. The “meet-in-the-middle” principle to show how biomarkers can be used prospectively to contribute to human health risk assessment and retrospectively in population-based studies to identify molecules for suitability as intermediate biomarkers of “‘early effect”effect’ to link exposure biomarkers with disease endpoints

*Biomonitoring* consists of standardized protocols for the periodic detection of early, preferably reversible, biological signs that are indicative, when compared with adequate reference values, of an actual or potential condition of exposure, effect or susceptibility, possibly resulting in health damage or disease. These signs are referred to as biomarkers [86]. In 1986, Henderson and Zielhuis defined the three types of biomarkers as biomarkers of exposure, of effect, and of susceptibility. Not all biomarkers can be easily classified in this system but it is useful to have a discussion as to how a specific biomarker can be effectively employed in a study design (see Additional file 1: Tables S6 and S7). The functional property of an exposure biomarker is that they carry the signature of a chemical/environmental contaminant marker that can be interpreted in the context of an exposure [87]. Effect biomarkers can provide information on the impact of environmental exposure on molecular, cellular or tissue levels. This ‘effect’ can be interpreted as an ‘adverse’ outcome but it often indicates a ‘response’ that can also be interpreted as a beneficial event, protecting the exposed individual, such as in enzymatic DNA repair. Thus, the terms “biomarker of response” or “‘biomarker of early effect” can also be used (see Fig. 6). A biomarker cannot always be attributed to a specific causative factor because most of these biomarkers lack chemical structural information, making them non-specific for the causative agent. The contextual information is important for making inferences about the possible involvement of one or more environmental exposures and the use of these biomarkers requires study formats that are particularly well thought-through in their design (Fig. 6).

Susceptibility biomarkers can be used to identify a person for a specific property that may be the result of genetic constitution, acquired properties or both. With these biomarkers, it is possible to classify an individual as “susceptible”‘or “resistant”‘to an exposure. The term “susceptibility”‘is also given a wide interpretation and, at a group level, it is possible to determine an effect measure, such as an Odds Ratio, to assess health risk for a subgroup with one (or more) susceptibility characteristics for comparison with another subgroup with a different susceptibility profile. It is not very useful to interpret a susceptibility outcome for an individual. As for the use of effect biomarkers, the interpretation of susceptibility biomarkers relies on a study designed to support interpretation of susceptibility data in a population.

Nowadays, biomarkers are often applied in population-based studies. Many years of experience have demonstrated that the laboratory-based analysis of biomarkers is usually performed well, because of well-established and rigorous quality assurance. Comparatively, other tasks are performed less successfully and are often suboptimal, e.g. sample collection and data interpretation may have weak spots that contribute to an overall moderate outcome (see Additional file 1: Table S8). As a consequence, biomonitoring studies sometimes do not provide useful results. In a recent study performed in response to the 9/11 Twin Towers terrorist attack in New York, the authors concluded that “‘*…*this study cannot provide any information about exposure or potential health effects” [88]. One of the problems was that insufficient consideration was given to the timing of sample collection relative to the time point of suspected exposure. Also, the groups that were selected for comparison of their biomarker levels were not well suited to the aims of the study.

The availability of a factsheet with the most relevant characteristics of a chemical substance may support well-informed decision-making during the preparation phase. During the EU FP7 project Biomonitoring of Exposure to Carcinogenic Substances (BIOMONECS), a format was developed for this purpose, with the biomonitoring application datasheets (BADS) providing the most relevant information in a concise format. For 15 chemical substances, BADS are available on www.humanbiologicalmonitoring.eu (last updated in 2010). The most recently published BADS are for mercury and methyl mercury [89]. This structured presentation may be useful if time is limited for the deployment of biomonitoring following a chemical incident.

The WG 2 group also supports the research and training of toxicology knowledge (including nanotechnology and particle toxicology, see below) and with knowledge about the management and risks of chemicals.

### Air pollution and particulate matter

#### Oxidative potential – a possible metric of particle toxicity

Air pollution is a complex mixture of chemically different components. Particulate matter (PM) has been designated as one of the most important components of the burden of disease from air pollution. How particles elicit their responses has not been fully elucidated but many studies have implicated the importance of reactive oxygen species (ROS) formation and oxidative stress in particle toxicity. The oxidative potential of a particles – their ability to generate ROS in cell- free systems – has been suggested as a promising metric for predicting particle toxicity. The oxidative potential could provide a simple screening tool for new nano-materials and a more relevant dose metric in epidemiological studies.

#### ROS and oxidative stress in particle-induced toxicity

**Fig. 7:**
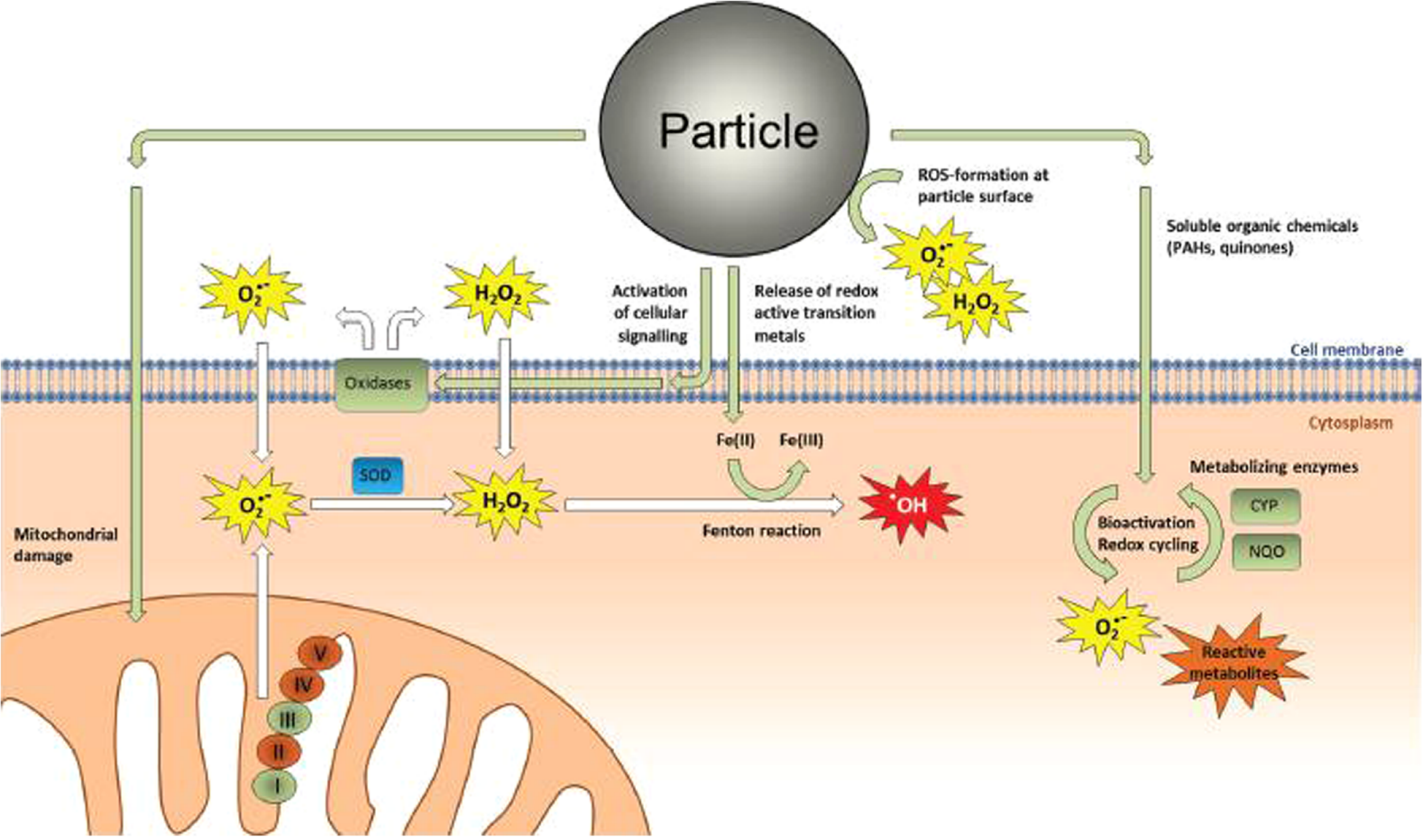
Potential sources of ROS formation in particle- exposed cells. *Note*: interpreting the effects of antioxidants on cellular responses from particle exposure is inherently difficult due to the many potential sources of ROS. ROS may be generated directly by reactive particle surfaces in contact with aqueous media, soluble organic constituents such as PAHs and quinones may form ROS and reactive electrophilic metabolites through redox cycling and metabolic activation, Fenton-reactive transition metals may contribute to formation of highly reactive hydroxyl radicals (●OH), activation of intracellular signaling pathways may trigger production of superoxide (O_2_●-) and hydrogen peroxide (H_2_O_2_) through activation of membrane bound oxidases, and damage to mitochondria may lead to superoxide production. The figure has previously been published in [95]

The role of ROS and oxidative stress in particle toxicology has been based, at least partly, on the observation that particles generate ROS in cell-free systems such as aqueous buffers, that increased ROS levels are measured within particle-exposed cells, and that antioxidants inhibit various cellular responses induced by particles [90–94]. Figure 7 presents an overview of cellular ROS production in particle-exposed cells [95] (see also Additional file 1: Table S9, [90–94, 96–100]). An overview of the endogenous components involved in cellular redox -regulation is presented in an attachment (Additional file 1: Table S10).

#### Beyond oxidative potential – the role of redox signaling in cellular responses to PM

Oxidative stress and redox responses are considered to play a central role in particle-induced toxicity. It is often assumed that the biological reactivity of a particle is because of its oxidative potential: the ability to produce ROS or be able to oxidize target substrates directly in contact with biological fluids or cellular molecules. However, particle exposure may also initiate a number of endogenous redox responses in cells or tissues. Inflammatory responses are considered to be contributory in the development or exacerbation of health effects from exposure to all types of particulates and they involve multiple levels of ROS-production and redox regulation. Clearly, inflammatory processes lead to oxidative stress, as activated immune cells produce and release ROS [96–99]. However, several pro-inflammatory genes are regulated through autocrine or paracrine signaling loops initiated by early cytokines such as interleukin (IL)-1α/β and tumor necrosis factor-α [101, 102]. Both the transcriptional activation and maturation/release of these pro-inflammatory mediators, as well as signaling from their respective receptors, involves multiple redox-regulated processes that could be affected by antioxidant treatment [91, 103–105]. The pathological importance of the oxidative potential of the particles versus these secondary redox responses of exposed cells may not be easy to disentangle, and may require much more sophisticated approaches than mere antioxidant treatment. An in-depth understanding of the role of endogenous redox regulation in these cellular responses is important, therefore, in order to clarify the relevance of oxidative potential as a metric for predicting particle toxicity.

### Rapid methods for the detection of disease/exposure biomarkers, infections, food and environmental contaminants

Previous sections have described the importance of detection of both hazardous materials and exposure-related markers for our understanding of the associated diseases and their development and for optimization of early detection and clinical interventions. This cannot be effectively achieved without high sensitivity, specific and multi-target analytical platforms that are robust and easy-to-use, can generate appropriate measurements very rapidly, and can be applied in non-laboratory settings. This approach will facilitate near-patient testing and is cognizant of the imperative to develop testing regimens that are accessible, minimally invasive and ameliorate the overburdening of health care services. This will require a variety of approaches and will definitely involve the integration of the measurement of different targets including proteins, miRNAs, circulating nucleic acids, cells, exosomes and many other molecular species. Thus, what is required is the capability to handle multiple matrices as sample sources, integration of binders to monitor specific targets (e.g. antibodies, nucleic acid probes) and highly sensitive detection strategies.

The focus of this DiMoPEx partner is the development of rapid diagnostic systems for the detection of a variety of targets using polyclonal, monoclonal and recombinant antibodies. The strategies used include electrochemical and optical detection (surface plasmon resonance, fluorescence, chemiluminescence and absorbance) mainly on microfluidics-based platforms. We generate recombinant antibody-derived structures highly customized for the specific application. We also run an international master’s program in biomedical diagnostics and are very involved in scientific approaches for antibody isolation, characterization and subsequent assay development.

In respect of outreach, a DiMoPEx training partner is the Applied Biochemistry Group, Dublin City University, which has a unit (AbYBiotech) that develops customized recombinant antibodies and has all the necessary equipment and developmental pipelines for antibodies and assays (see Additional file 1: Table S11). The successful antibodies need to be fully characterized, with the entire sequence defined and published or available, and this is now facilitated through the use of recombinant antibodies. In relation to DiMoPEx, the group provides opportunities for collaboration in a number of areas, including antibody generation, assay use and validation, exchange of researchers for training, development of education and science outreach. Examples of the research and the potential for collaboration within DiMoPEx can be gleaned from the literature published by the group [106–112].

## WG 3: Environmental and occupational epidemiology overarching other WGs

Epidemiology is the branch of science that deals with the study of the causes, distribution, and control of disease in populations. Occupational and environmental epidemiology deals specifically with the impact of occupational and environmental exposures on health and disease in populations. Although experimental and toxicological methods to establish mechanisms for a certain exposure and its impact on organisms are available, most often the only way to confirm the link between an exposure and the outcome is observational epidemiologic studies addressing disease occurrence in human populations. Such population-based studies are the only way to address the exposure-response relationship and to explore susceptibility and societal exposures simultaneously.

Radon exposure, for example, affects smokers and non-smokers equally but, because smokers have a higher basic risk, the total burden is dominated by lung cancer in smokers. As an example, the International Agency for Research on Cancer (IARC) evaluates whether a certain exposure can cause cancer in humans based on epidemiological evidence or a combination of evidence from epidemiological and animal or mechanistic studies (www.IARC.fr).

The objectives of the Epidemiology WG (WG3) are to:

- provide a sustainable research and training program in the field of environmental and occupational epidemiology for early career investigators;
- In collaboration with other WGs, provide opportunities for participation in environmental and occupational epidemiological research, more specifically in the development of novel exposure–response relationships, in the area of exposure-related diseases;
- provide training in the epidemiology of exposure-related diseases – computer skills training will focus on epidemiological modelling. Including spatio-temporal, exposure-response and interaction modelling.

In DiMoPEx, WG3 provides input and expertise with regards to study design for the other WGs. In collaboration with WG1 and WG2 for example, the WG3 contributes with knowledge about exposure assessment strategies and how to utilize the available data in the most efficient way by exploring alternative exposure metrics (for example, individual measurements versus group-based measurements, cumulative exposure versus period-specific exposure). The members of WG3 share experience with the use of large register-based population studies, in combination with individual exposure measurements obtained from industry-based cohort studies, in order to learn about the advantage of both types of data. WG3 includes researchers with experience in different kinds of exposure-assessment tools in individuals, groups or large populations, including biomarkers, individual- and area- based exposure measures in the environment, and questionnaires. Figure 2 displays the major tools for exposure assessment.

We have introduced register data sources from Denmark – (Danish Occupational Cohort DOC*X (www.DOC-X.dk) and register resources at Aarhus University (http://cirrau.au.dk/). Furthermore, in collaboration with other WGs, the WG Epidemiology WG3 group has submitted a number of spin-off research project proposals, including “Can pyrethroid pesticides cause diabetes?” and “The effect of organic dust and endotoxin exposure on CVD, lung cancer and interstitial lung disease”.

## WG 4 provides solutions for ethical aspects of data collection and communication for other groups

WG 4 supports the research of other groups and provides training programs for all ethical aspects of data collection, communication and publication ethics. Focusing on early carrier investigators (which are in focus of COST program actions), the DiMoPEx project aims to provide knowledge about data privacy regulations.

### Ethics framework

An international ethics reference framework for biomedical research involving human subjects already exists and the researchers can and must be able to refer to this in their work (see Additional file 1: Table S12). While this reference framework is international, the legal anchoring of principles to which this framework refers is provided by national law. National regulations are mostly based on the Oviedo Convention and its Additional Protocol on biomedical research that emphasize: the necessity of obtaining informed consent, the requirement that a research project is submitted to an ethics committee for independent examination of its scientific merit and multidisciplinary review of its ethical acceptability (in each country in which any research activity is to take place).

Some challenges must be considered carefully: the authenticity of informed consent, data protection as a possible obstacle for research, the secondary use of data/samples, the right of an individual to know or not to know, and dealing with communication at a collective level.

#### Informed consent

In general, the authenticity of informed consent can be questioned for several reasons.:

- The authority or status of the person providing the information may decisively affect the outcome.
- The accuracy of the information provided can be limited.
- Correct understanding of the information is a prerequisite and cannot be assumed if not checked.
- The right to decide is not always synonymous with the ability to decide for oneself.
- Decisional autonomy can be in conflict with social constraints.
- The consequences of a decision may be affected by a perception of power inequality, for instance, when access to a right can be denied as a consequence of the outcome.

The process of obtaining informed consent is the outcome of a complex interaction of personalities. Awareness and understanding is necessary for correctly implementing the process. In a pragmatic way, one may consider consent as authentic when the person is: clearly free in the decision to participate, is equal in relationship with the recruiter, is listened to and receives answers at his or her personal level of understanding, and comprehends what s/he consents to.

#### Communication/right-to-know

The research subjects might have a legally embedded right to know their individual results from the research, if they wish. IIndividual results are often not provided to the study participants because of:

- lack of relevance of the results at an individual level,
- limited time and/or resources,
- fear of causing (unnecessary) alarm,
- scientific uncertainty,
- lack of possible remediation.

#### Participatory (community engagement) approaches

From an ethical perspective and from a perspective of increasing confidence and trust in researchers and their research, it is often not sufficient to leave the decision to participate in a study to every single individual. The involvement of community members or representatives of the relevant community in consultation, as a complement to decision-making autonomy, may also be needed. This requires the development of methods to include community consultation, community-based participatory research, and community consent to research. This can be done through processes of cooperative inquiry.

#### Communication

Spreading information about research outcomes is essential and must occur at the individual as well as at the collective level. Sufficient information is necessary at recruitment, during the study, and while disseminating results (individual and collective, including policy).

There are many challenges for protecting human dignity and the right of the individual research participant, whilst at the same time not hampering the progress of research. Practices show a strong belief in scientific work. Societal acceptance of practices will depend on good communication at all levels. The future of research with human subjects will, to a large extent, depend upon the trust and confidence which is generated in the perception of these (potential) research participants.

#### Human data sampling and collection: Imminent new OECD and EU data privacy regulations

The last few years have witnessed an important expansion of human DNA sampling and data collecting in order to exploit and study the genetic information collected. The strategic importance of this activity for genetic research and its applications is obvious, yet many DNA banks are concerned about how to obtain valid informed consent and how to deal with retrospective collections (see Additional file 1: Info Box 5, on new OECD, Global Science Forum and on EU-The General Data Protection Regulation, which will become law across the EU in May 2018).

## WG 6 steps towards NCD diagnosis and monitoring

Since NCDs not only cause premature deaths and increased morbidity but also have a significant economic impact, the cost-effective and evidence-based interventions and tools to prevent and control various NCDs must include:

- reduction of causative exposures/risk factors;
- early detection and management of respective disorders;
- surveillance of endangered populations to monitor trends in risk factors and diseases (WG6 in cooperation with other WGs).

Such interventions are feasible, but they do necessitate a paradigm shift, away from considering each singular exposure towards the addressing disease clusters collectively in an integrated manner (“exposome”), moving away from a purely clinical approach towards a fully integrated public health approach.

An integrated approach targeting all major common risk factors, with the aim of reducing premature mortality and morbidity of chronic NCDs, is clearly the most cost-effective way to prevent and control the common NCDs. This requires the integration of primary, secondary, and tertiary prevention, health promotion and related programs across numerous sectors and different disciplines. In order to enhance interdisciplinary cooperation, a clinical network concentrating on exposure-related diseases will work with DiMoPEx partners in order to 1) develop common diagnostic scheme guidelines to aid physicians and public health workers to make best use of the evidence; and 2) integrate NCDs intervention initiatives in the health system based on primary health care. The interdisciplinary team of young European researchers will have the opportunity to use the analyses within the framework of the DiMoPEx project to generate risk assessment and prevention models to improve health and safety in Europe for the general public, and, more specifically, for workers and consumers.

### Current human studies applying outlined methods on various exposure scenarios

#### Exposure to welding fumes and cardiovascular toxicity

DiMoPEx partner, Unit Metals and the Health Unit of the Institute of Environmental Medicine, Karolinska Institutet, Sweden, is currently performing research on health problems in the work environment (welding fumes and exposure to soot particles) and early-life exposure to metals and health effects during childhood). The projects are described in brief below:

Exposure to welding fumes increases the risk of CVD and workplace exposure to welding particles occurs frequently in Sweden and worldwide. However, we still do not know what levels of exposure are sufficient to increase the risk of CVD, and whether current welders remain at increased risk ot not. In 2010, the group enrolled welders and controls, all male non-smokers, in southern Sweden, who were characterized for exposure to particles and received medical examinations. The authors found that low-to-moderate exposure to welding fumes can be a risk factor for hypertension [113, 114]. Moreover, the data indicate that welding fumes cause premature ageing of the cardiovascular system [115], possibly by increasing oxidative stress [114, 115] from the high metal content of the welding fumes, as well as epigenetic changes of the F2RL3 gene, a CVD marker [116].

In contrast, the authors did not find signs of other previously suggested mechanisms for cardiovascular damage involving exposure to particles [112]. Our group is now re-examining welders and controls to validate our cross-sectional findings and quantify the effects of welding particle exposure on the cardiovascular system, as well as to explore mechanisms of action, by using a longitudinal approach. The information about medical and occupational histories from the welders and controls is being collected and their heart-rate variability and endothelial function measured. Further, blood and urine samples are being collected for measurement of markers of premature ageing and oxidative stress, as well as markers of inflammation and one-carbon metabolism and of coagulation. The approach will address novel hypotheses, help explain findings from previous studies, assist in risk assessment, and improve advice to welders on the safety of working with welding fumes.

#### Chimney sweeping and risk of cancer

Chimney sweeps in Sweden have an excess risk of bladder, liver, lung and esophagus cancer. The increase in risk is likely due to exposure to polycyclic aromatic hydrocarbons (PAHs) [117]. It is necessary, therefore, to clarify to what extent contemporary Swedish chimney sweeps exhibit cancer-related DNA changes and if current levels of PAH exposure are genotoxic. 1-hydroxypyrene (1-OHP) has traditionally been used as a proxy for total PAH exposure, although 1-OHPHP is itself not carcinogenic. A more relevant marker of carcinogenic PAHs, but much less studied, is 3-hydroxybenzo(a)pyrene (3-OHBaP) [118]. The aim of the study is to determine early carcinogenic DNA changes in Swedish chimney sweeps and to investigate the association between current exposure and genotoxicity. Chimney sweeps have been recruited for determining exposure, for medical examinations and for the sampling of biomarkers of DNA damage in blood and urine. Biomarkers and medical information are also collected in a control set of male warehouse workers with low exposure to PAHs.

The study will clarify whether current exposure experienced by chimney sweeps is carcinogenic. If there are stronger associations for 3-eOHBaP than for 1-OHP with genotoxicity, this may affect methods used for risk assessment of PAHs in general, which is important for the workplace as well as for the general population.

#### Pesticides exposure and GxE testing in Parkinson’s disease

Parkinson’s disease (PD) is a chronic progressive neurodegenerative movement disorder that affects 1% of the population over the age of 60 years and both genetic and environmental factors contribute to its etiology. Specifically, occupational pesticide exposures have been identified as risk factors for PD, but the quality of exposure assessment varies considerably between studies with only a few identifying exposure to specific chemicals.; Some studies recorded ever/never occupational exposure or self-reports of specific pesticides [119] while others created job exposure matrixes (JEMs) [120–125], and only one –- the USA Agricultural Health Study (AHS) cohort of licensed pesticide applicators –- used a prospective design and collected specific pesticide use in great detail [126]. Our own California study (known as the Parkinson’s, Environment and Genes PEG study) recently provided some of the strongest evidence yet that specific pesticides in combination with genetic susceptibility contribute to the etiology of PD in humans and that certain pesticides affect pathogenic pathways that have been related to neurodegeneration. In this population-based case control study that was conducted in the heavily agricultural central valley of California, [127], detailed historical data for active occupational and household pesticide use was collected and, most importantly, we were able to employ a geographic information system to assess ambient pesticide exposures from agricultural applications at workplaces and residences. To generate these exposures to pesticides, we were able to rely on the state-mandated California Pesticide Use Reporting (PUR) system (active since 1974), digitized historical land-use maps and address histories of the participants [128]. Combining these data sources, we pinpointed pesticide applications at a precise agricultural site and related these to the home and work addresses of the participants to calculate time-specific pesticide exposures based on application rates per acreage or pounds of pesticide per acre applied annually in the proximity of their homes or workplaces. Using this unique exposure assessment tool and the data and bio-samples collected from almost 1800 study participants, the PEG study provided the first human evidence that a specific combination exposure (paraquat and maneb) increased the risk of PD, confirming animal model findings [129] from toxicological research. We also found that both residential and workplace exposures contribute to PD risk [130], as did household use of organophosphate pesticides [131] and consumption of contaminated well-water [132]. Importantly, we identified gene-environment interactions for genes in molecular pathways that contribute to PD pathology according to animal/ cell studies. The major pathophysiologic mechanisms we addressed included: 1) dopamine transporter activity, (*DAT*); dopamine metabolism pathways (aldehyde dehydrogenase 1 family, member A1 gene -*ALDH1* [133]) relevant to PD; and mitochondrial dysfunction due to oxidative/nitrosative stress (nitric oxide synthase 1 (neuronal) gene – n***NOS)*** [134]. We also identified genetic susceptibility in the proteasomal pathways (***SKP1***-gene [135]), especially when combined with exposure to proteasome-inhibiting pesticides (di-thiocarbamates): genetic susceptibility related to the response of the innate immune system among those exposed to pyrethroid pesticides (MHC class II cell surface receptor encoded by the human leukocyte antigen - ***HLA-DR)*** [136]; DNA repair gene variants (DNA (apurinic or apyrimidinic site)-lyase gene, 8-Oxoguanine glycosylase 1 (DNA glycosylase) gene, ***APEX1, OGG1*** [136]) that affect mitochondrial function via oxidative stress; and, finally, genetic susceptibility to the neurotoxic action of organophosphate pesticides for carriers of variants in the pesticide metabolism gene serum paraoxonase/arylesterase1, ***PON1*** [137]). A summary of our findings has been published recently in the journal *Current Environmental Health Reports* [127], in which the importance of integrating genetic information with advanced exposure assessment methods to describe the combined impact of genes and environment on biologic pathways relevant to disease was praised.

Further joint research projects from DiMoPEx partners that focus on the impact of the pollution on human health are presented in reference [138].

## WG 7: Public health protection – how to stimulate interaction between scientist and policy makers

### Collaboration with WHO

In the WHO European Region, diabetes, CVD, cancer, chronic respiratory diseases and mental disorders cause no less than 86% of deaths and 77% of the disease burden, with marked inequalities reflecting a social gradient (WHO, 2012, see Additional file 1: Info Box 1). The regional strategy and action plan, frame prevention and control efforts do focus on the proximal risk factors, while acknowledging the relevance of environmental and occupational factors (WHO, 2012, see Additional file 1). DiMoPEx partner(s) aim to collaborate with the WHO, focusing on environmental determinants of health (with a spotlight on chemical, fume and dust exposures in living and working environments). Of mutual interest, is the analysis of and action on modifiable environmental conditions or their modifiable components (http://dimopex.eu/ncds/). In the context of human health and disease, consideration of the environment focuses on the aspects that can be modified through intervention, leading to reduced human exposure and health impacts, hence offering opportunities for preventative measures. When assessing the GBD, WHO analyses modifiable environmental conditions, including: pollution of air, water or soil by chemical or biological agents; occupational risks; ultraviolet and ionizing radiation; noise; electromagnetic fields; built environments and housing; land-use patterns; roads; major infrastructural and engineering works (roads, dams, railways); agricultural methods; irrigation schemes; man-made vector breeding places; climate and ecosystem change; environment-related behavior (WHO, 2016, see Additional file 1).

A resolution on the health impact of air pollution, adopted at the Sixty-eighth World Health Assembly in May 2015, and a road map for an enhanced global response to air pollution by the health sector provide a framework to guide actions by Member States, WHO and stakeholders globally. In the WHO European Region, the Health 2020 policy, as well as policy commitments from the Sixth Ministerial Conference on Environment and Health (Ostrava, June 2017), combine to guide regional efforts designed to reduce environmental burdens on health and to promote environment-related health benefits (WHO, 2013; WHO, 2017, see Additional file 1). Occupational risks contribute to the burden of NCDs (WHO, 2016, see Additional file 1). The burden of disease because of occupational risk factors, estimated by the GBD project group, included 304,000 deaths from occupational carcinogens (largely asbestos), 205,000 deaths from occupational PM, gases and fumes, with 52,000 deaths from occupational asthmogens (GBD 2013 Risk Factors Collaborators, 2015, see Additional file 1). Important occupational diseases induced by mineral dust and fiber exposure are pneumoconioses. This group of chronic respiratory diseases, including silicosis, asbestosis and coal-workers’ pneumoconiosis, is estimated to cause 260,000 deaths per year globally (GBD 2013 Mortality Causes of Death Collaborators, 2015, in WHO (2016, see Additional file 1). Since these NCDs are also our focus, the DiMoPEx partners intend to implement the methodological approaches from WHO and contribute to the process of producing an estimate of the environmental burden of diseases. The common approaches include: comparative risk assessment, calculations based on epidemiological data and expert opinion to fill current gaps in knowledge.

### Dissemination and implementation of new knowledge within a scientific network

The DiMoPEx COST Action is dedicated to catalyze a joint effort of European scientists to address the issue of adverse health effects of environmental exposures and to suggest ways of evaluating and managing them. The WG 7 is committed to these goals. In this process, facilitation and coordination of information transfer among the participants, such as between the action core group and external partners, and effective wide-scale dissemination and implementation of the new knowledge produced by the project are essential features.

The first opportunity for networking and for exchange of knowledge and ideas was the combined meeting of DiMoPEx WGs in Hamburg in June 2016, when participants had the opportunity to present their expertise and backgrounds using posters and thematic oral presentations. The second working groups meeting was in Bentivoglio, Italy in October 2017 [138]. In future, specific WG meetings will serve the purpose of formulating concrete plans for joint projects between the partners and affiliates and will prepare the ground for formulating new projects. Another important tool of internal knowledge dissemination is the organization of training schools (e.g. on exposure assessment, occupational and environmental epidemiology, MN methods) and short-term scientific missions of individual institutional and laboratory visits that provide an opportunity for building capability in early career investigators, primarily.

The involvement of external partners in the activities of the Action, such as the European Society for Environmental and Occupational Medicine, the Collegium Ramazzini (http://www.collegiumramazzini.org/about.asp) and the WHO European Centre for Environment and Health, is an important priority. In collaboration with WHO and the International Labour Organization, the DiMoPEx partners perform a systematic review of the relationship between pneumoconiosis and occupational dust and fiber exposures, the results of which allow the estimation of the related burden of disease.

The DiMoPEx website serves as the main platform for informing participants (http//dimopex.eu), external partners, and the wider-scale scientific and decision-making community about the research backgrounds of the participants, plans for cooperation, events, activities, grant applications, formulating and ongoing projects, and results that can be related to the Action.

A further goal of the WG 7 is assembling and critically assessing information, creating new knowledge, and implementing this knowledge, by testing and formulating feasible recommendations for the evaluation and management of health risks of environmental exposures and publishing the results in various electronic and printed media. Apart from the scientific community, the decision- makers of topic-related sectoral policies and industries, as well as the general public, are considered important targets for the dissemination of DiMoPEx results. The research community is primarily informed through peer-reviewed scientific publications, research articles, textbooks and guidelines. At the end of the COST-Action, a conference will be organized that will address not only scientists but also decision- makers and the general public, with sessions directed to them appropriately, and the relevant messages will be disseminated by an appropriate media coverage.

To ensure the effective and sustainable implementation of the new knowledge produced, tailored information will directly be delivered to decision-makers by printed and electronic leaflets and via the DiMoPEx website.

## Summary and conclusions

- Environmental hazardous exposure is among the top risk factors for chronic disease mortality. A better understanding of the health-environment (including the gene-environment) and its interactions in the etiology of NCDs allows more adequate preventative actions that could decrease disease morbidity and mortality for many of the NCDs that are of major public concern.
- Within the COST action DiMoPEx, models will be developed for the assessment of hazardous exposures and their potential health consequences using collected data and available toxicological/epidemiological evidence.
- DiMoPEx partners believe that combining state-of- the-art exposure assessment methods with clinical efforts should grant a more solid basis for both early recognition and diagnosis strategies, as well as for the advancement of preventive strategies in Europe.
- The predominant goals of the DiMoPEx project arinclude helping scientists, physicians and health officials in preventing and reducing health impairments associated with various exposure scenarios and to train highly researchers in these disciplines with the requisite skills.
- Risk communication expertise developed within the DiMoPEx action and tools to inform exposed subjects and the general public are expected to benefit society.

## Additional file

Additional file 1: Info Box 1–-3. Tables 1–-11. Including additional references ([139–153]). (DOCX 504 kb)

## Abbreviations

1-OHP: 1-hydroxypyrene
AHS: Agriculatural Health Study
ALDH1: Aldehyde dehydrogenase 1 family, member A1 gene
APEX1: DNA-(apurinic or apyrimidinic site) lyase gene
BADS: Biomonitoring application datasheets
CBMN: Cytokinesis-block micronucleus
cfDNA: Circulating free DNA
CMCRC: Cesare Maltoni Cancer Research Center
COST: EU programme, European Cooperation in Science and Technology
CVD: Cardiovascular disease
DAT: Dopamine transporter activity
DiMoPEx: Diagnosis, Monitoring and Prevention of Exposure-related Noncommunicable Diseases
DNA: Desoxyribonucleic acid
EMM: Effect measure modifications
EUEFSA: European Union Food safety Authority
GBD: Global Burdenburden of Disease (study)diseases
HLA-DR: MHC class II cell surface receptor encoded by the human leukocyte antigen
HP: Hydoxypyrene
IARC: International Agency for Research on Cancer
IARC: International Agency for Research on Cancer
ILO: International labour organization
JEM: Job exposure matrix
miRNA: micro RNA
MN: Micronucleus
NCD: Noncommunicable diseasenon-communicable diseases
nNOS1: Nitric oxide synthase 1 (neuronal) gene
NOEL: No Observed Adverse Effect Level
OECD: OrganisationOrganization for Economic Co-operationCooperation and Development
OGG1: 8-Oxoguanine glycosylase 1 (DNA glycosylase) gene
PAH: Polycyclic aromatic hydrocarbons
PD: Parkinson’s disease
PEG: Parkinson’s Environment and Genes Study
PM: Particulate matter
PON1: Gene coding for serum, Serum paraoxonase/arylesterase 1
PUR: California Pesticide Use Reporting
RI: Ramazzini Institute
RNA: RibonucleicRibonucleic acid
ROS: Reactive oxygen species
SD: Sprague-Dawley (rats)
TNF: Tumor necrosis factor
UTR: Untranslational region
WG: Working group
WHO: World Health Organization

## References

[1] World Health Organization. Global health risks: mortality and burden of disease attributable to selected major risks. http://www.whoint/healthinfo/global_burden_disease/GlobalHealthRisks_report_fullpdf 2009.

[2] Centers of Disease Control and Protection (CDC). Global Health Protection and Security: Global Noncommunicable Diseases (NCDs). http://www.cdcgov/globalhealth/healthprotection/ncd/ 2017.

[3] Lim SS, Vos T, Flaxman AD, Danaei G, Shibuya K, Adair-Rohani H, Amann M, Anderson HR, Andrews KG, Aryee M (2012). A comparative risk assessment of burden of disease and injury attributable to 67 risk factors and risk factor clusters in 21 regions, 1990-2010: a systematic analysis for the Global Burden of Disease Study 2010. Lancet.

[4] World Health Organization: 7 million premature deaths annually linked to air pollution. http://www.whoint/mediacentre/news/releases/2014/air-pollution/en/ 2015.

[5] Landrigan PJ, Fuller R, Acosta NJR, Adeyi O, Arnold R, Basu NN, Balde AB, Bertollini R, Bose-O'Reilly S, Boufford JI, et al. The Lancet Commission on pollution and health. Lancet. 2017; 10.1016/S0140-6736(17)32345-0 29056410

[6] WHO: The cost of a polluted environment: 1.7 million child deaths a year. http://www.whoint/mediacentre/news/releases/2017/pollution-child-death/en/ 2017.

[7] The WHO Global Health Estimates. http://www.whoint/healthinfo/global_burden_disease/en/ 2014.

[8] Institute for Health Metrics and Evaluation (IHME), Seattle, WA, USA. http://www.healthdataorg/data-visualization/gbd-compare 2017. 10.1016/S0140-6736(17)30263-5 28170325

[9] Raaschou-Nielsen O, Andersen ZJ, Beelen R, Samoli E, Stafoggia M, Weinmayr G, Hoffmann B, Fischer P, Nieuwenhuijsen MJ, Brunekreef B (2013). Air pollution and lung cancer incidence in 17 European cohorts: prospective analyses from the European Study of Cohorts for Air Pollution Effects (ESCAPE). Lancet Oncol.

[10] Burnett RT, Pope CA, Ezzati M, Olives C, Lim SS, Mehta S, Shin HH, Singh G, Hubbell B, Brauer M (2014). An integrated risk function for estimating the global burden of disease attributable to ambient fine particulate matter exposure. Environmental health perspectives.

[11] Rappaport SM (2011). Implications of the exposome for exposure science. J Expo Sci Environ Epidemiol.

[12] Rappaport SM, Smith MT (2010). Epidemiology. Environment and disease risks. Science.

[13] Manthripragada AD, Costello S, Cockburn MG, Bronstein JM, Ritz B (2010). Paraoxonase 1, agricultural organophosphate exposure, and Parkinson disease. Epidemiology.

[14] Ritz BR, Manthripragada AD, Costello S, Lincoln SJ, Farrer MJ, Cockburn M, Bronstein J (2009). Dopamine transporter genetic variants and pesticides in Parkinson's disease. Environmental health perspectives.

[15] Blair A, Ritz B, Wesseling C, Freeman LB (2015). Pesticides and human health. Occup Environ Med.

[16] Hertz-Picciotto I, Smith AH, Holtzman D, Lipsett M, Alexeeff G (1992). Synergism between occupational arsenic exposure and smoking in the induction of lung cancer. Epidemiology.

[17] Budnik LT, Austel N, Gadau S, Kloth S, Schubert J, Jungnickel H, Luch A (2017). Experimental outgassing of toxic chemicals to simulate the characteristics of hazards tainting globally shipped products. PloS one.

[18] Budnik LT, Wegner R, Rogall U, Baur X (2014). Accidental exposure to polychlorinated biphenyls (PCB) in waste cargo after heavy seas. Global waste transport as a source of PCB exposure. International archives of occupational and environmental health.

[19] Budnik LT, Kloth S, Baur X, Preisser AM, Schwarzenbach H (2013). Circulating mitochondrial DNA as biomarker linking environmental chemical exposure to early preclinical lesions elevation of mtDNA in human serum after exposure to carcinogenic halo-alkane-based pesticides. PloS one.

[20] Sass J, Heine L, Hwang N (2016). Use of a modified GreenScreen tool to conduct a screening-level comparative hazard assessment of conventional silver and two forms of nanosilver. Environmental health : a global access science source.

[21] Weisel CP (2002). Assessing exposure to air toxics relative to asthma. Environmental health perspectives.

[22] Abrahamsen R, Fell AK, Svendsen MV, Andersson E, Toren K, Henneberger PK, Kongerud J (2017). Association of respiratory symptoms and asthma with occupational exposures: findings from a population-based cross-sectional survey in Telemark. Norway. BMJ Open.

[23] Thurston GD, Kipen H, Annesi-Maesano I, Balmes J, Brook RD, Cromar K, De Matteis S, Forastiere F, Forsberg B, Frampton MW, et al. A joint ERS/ATS policy statement: what constitutes an adverse health effect of air pollution? An analytical framework. The European respiratory journal. 2017;49 10.1183/13993003.00419-2016 PMC5751718 28077473

[24] Brauer M, Amann M, Burnett RT, Cohen A, Dentener F, Ezzati M, Henderson SB, Krzyzanowski M, Martin RV, Van Dingenen R (2012). Exposure assessment for estimation of the global burden of disease attributable to outdoor air pollution. Environ Sci Technol.

[25] CDC. National Center for Health Statistics: Leading Causes of Death. http://www.cdcgov/nchs/fastats/leading-causes-of-deathhtm 2017.

[26] Soffritti M, Belpoggi F, Minardi F, Maltoni C (2002). Ramazzini Foundation cancer program: history and major projects, life-span carcinogenicity bioassay design, chemicals studied, and results. Annals of the New York Academy of Sciences.

[27] World Health Organization. Environmental Health Criteria 214. Human exposure assessement. International Programme on Chemical Safety. http://www.inchemorg/documents/ehc/ehc/ehc214htm 2000.

[28] Den Hond E, Paulussen M, Geens T, Bruckers L, Baeyens W, David F, Dumont E, Loots I, Morrens B, de Bellevaux BN (2013). Biomarkers of human exposure to personal care products: results from the Flemish Environment and Health Study (FLEHS 2007-2011). Sci Total Environ.

[29] Huang L, Ernstoff A, Fantke P, Csiszar SA, Jolliet O (2017). A review of models for near-field exposure pathways of chemicals in consumer products. Sci Total Environ.

[30] Ginsberg GL, Balk SJ (2016). Consumer products as sources of chemical exposures to children: case study of triclosan. Curr Opin Pediatr.

[31] Steiling W, Bascompta M, Carthew P, Catalano G, Corea N, D'Haese A, Jackson P, Kromidas L, Meurice P, Rothe H, Singal M (2014). Principle considerations for the risk assessment of sprayed consumer products. Toxicology letters.

[32] Villanueva CM, Kogevinas M, Cordier S, Templeton MR, Vermeulen R, Nuckols JR, Nieuwenhuijsen MJ, Levallois P (2014). Assessing exposure and health consequences of chemicals in drinking water: current state of knowledge and research needs. Environmental health perspectives.

[33] Hernandez AF, Tsatsakis AM (2017). Human exposure to chemical mixtures: Challenges for the integration of toxicology with epidemiology data in risk assessment. Food Chem Toxicol.

[34] Cachada A, da Silva EF, Duarte AC, Pereira R (2016). Risk assessment of urban soils contamination: The particular case of polycyclic aromatic hydrocarbons. Sci Total Environ.

[35] Sobus JR, DeWoskin RS, Tan YM, Pleil JD, Phillips MB, George BJ, Christensen K, Schreinemachers DM, Williams MA, Hubal EA, Edwards SW (2015). Uses of NHANES Biomarker Data for Chemical Risk Assessment: Trends, Challenges, and Opportunities. Environmental health perspectives.

[36] Dorne JL (2010). Metabolism, variability and risk assessment. Toxicology.

[37] Valcke M, Krishnan K (2014). Characterization of the human kinetic adjustment factor for the health risk assessment of environmental contaminants. Journal of applied toxicology : JAT.

[38] Tennekes HA, Sanchez-Bayo F (2013). The molecular basis of simple relationships between exposure concentration and toxic effects with time. Toxicology.

[39] OECD. Test No. 453: Combined Chronic Toxicity/Carcinogenicity Studies. OECD Guidelines for the Testing of Chemicals. Section 4: Health Effects. France: OECD Publishing Paris; 2009. http://www.oecd-ilibrary.org/environment/test-no-453-combined-chronic-toxicity-carcinogenicity-studies_9789264071223-en.

[40] OECD Test No. 443: Extended One-Generation Reproductive Toxicity Study. OECD Guidelines for the Testing of Chemicals, Section 4: Health Effects. . OECD Publishing Paris, France 2011. http://www.oecd.org/env/test-no-443-extended-one-generation-reproductive-toxicity-study-9789264122550-en.htm.

[41] OECD Guideline for the testing of chemicals. Combined Chronic toxicity/carcinogenicity studies. TG 453. 2009.

[42] Teitelbaum SL, Li Q, Lambertini L, Belpoggi F, Manservisi F, Falcioni L, Bua L, Silva MJ, Ye X, Calafat AM, Chen J (2016). Paired Serum and Urine Concentrations of Biomarkers of Diethyl Phthalate, Methyl Paraben, and Triclosan in Rats. Environmental health perspectives.

[43] Silvestri F, Bussani R, Giarelli L (1991). Changes in underlying causes of death during 85 years of autopsy practice in Trieste. IARC Sci Publ.

[44] Maltoni C, Ciliberti A, Pinto C, Soffritti M, Belpoggi F, Menarini L (1997). Results of long-term experimental carcinogenicity studies of the effects of gasoline, correlated fuels, and major gasoline aromatics on rats. Annals of the New York Academy of Sciences.

[45] National Toxicology Program.Toxicology and carcinogenesis studies of xylenes (mixed) (cas no. 1330-20-7) in F344/N rats and B6C3F1 mice (gavage studies). https://www.ncbinlmnihgov/pubmed/12732897 1986. 12732897

[46] Belpoggi F, Soffritti M, Guarino M, Lambertini L, Cevolani D, Maltoni C (2002). Results of long-term experimental studies on the carcinogenicity of ethylene-bis-dithiocarbamate (Mancozeb) in rats. Annals of the New York Academy of Sciences.

[47] Manservisi F, Marquillas CB, Buscaroli A, Huff J, Lauriola M, Mandrioli D, Manservigi M, Panzacchi S, Silbergeld EK, Belpoggi F (2017). An Integrated Experimental Design for the Assessment of Multiple Toxicological End Points in Rat Bioassays. Environmental health perspectives.

[48] Bonassi S, Znaor A, Ceppi M, Lando C, Chang WP, Holland N, Kirsch-Volders M, Zeiger E, Ban S, Barale R (2007). An increased micronucleus frequency in peripheral blood lymphocytes predicts the risk of cancer in humans. Carcinogenesis.

[49] Maluf SW, Erdtmann B (2001). Genomic instability in Down syndrome and Fanconi anemia assessed by micronucleus analysis and single-cell gel electrophoresis. Cancer Genet Cytogenet.

[50] Bonassi S, El-Zein R, Bolognesi C, Fenech M (2011). Micronuclei frequency in peripheral blood lymphocytes and cancer risk: evidence from human studies. Mutagenesis.

[51] Migliore L, Coppede F, Fenech M, Thomas P (2011). Association of micronucleus frequency with neurodegenerative diseases. Mutagenesis.

[52] Andreassi MG, Barale R, Iozzo P, Picano E (2011). The association of micronucleus frequency with obesity, diabetes and cardiovascular disease. Mutagenesis.

[53] Baudhuin LM, Donato LJ, Uphoff TS (2012). How novel molecular diagnostic technologies and biomarkers are revolutionizing genetic testing and patient care. Expert Rev Mol Diagn.

[54] Schwarzenbach H, Hoon DS, Pantel K (2011). Cell-free nucleic acids as biomarkers in cancer patients. Nat Rev Cancer.

[55] Stroun M, Maurice P, Vasioukhin V, Lyautey J, Lederrey C, Lefort F, Rossier A, Chen XQ, Anker P (2000). The origin and mechanism of circulating DNA. Ann N Y Acad Sci.

[56] Mittra I, Nair NK, Mishra PK. Nucleic acids in circulation: are they harmful to the host? Biosci. 2012;37(2):301-12. 10.1007/s12038-012-9192-8 22581336

[57] Chen X, Liang H, Zhang J, Zen K, Zhang CY (2012). Horizontal transfer of microRNAs: molecular mechanisms and clinical applications. Protein & cell.

[58] Schwarzenbach H. Circulating nucleic acids in early diagnosis, prognosis and treatment monitoring: an introduction. Section VII: CNAPS and General Medicine. Holland: Springer Science+Business Media Dordrecht; 2015. p. 143-163.

[59] Dong H, Lei J, Ding L, Wen Y, Ju H, Zhang X (2013). MicroRNA: function, detection, and bioanalysis. Chem Rev.

[60] Reid G, Kirschner MB, van Zandwijk N (2011). Circulating microRNAs: Association with disease and potential use as biomarkers. Crit Rev Oncol Hematol.

[61] Pant S, Hilton H, Burczynski ME (2012). The multifaceted exosome: biogenesis, role in normal and aberrant cellular function, and frontiers for pharmacological and biomarker opportunities. Biochem Pharmacol.

[62] Zhao L, Liu W, Xiao J, Cao B (2015). The role of exosomes and "exosomal shuttle microRNA" in tumorigenesis and drug resistance. Cancer letters.

[63] Schwarzenbach H (2015). The clinical relevance of circulating, exosomal miRNAs as biomarkers for cancer. Expert Rev Mol Diagn.

[64] Rozek LS, Dolinoy DC, Sartor MA, Omenn GS (2014). Epigenetics: relevance and implications for public health. Annu Rev Public Health.

[65] Chappell G, Pogribny IP, Guyton KZ, Rusyn I (2016). Epigenetic alterations induced by genotoxic occupational and environmental human chemical carcinogens: A systematic literature review. Mutat Res Rev Mutat Res.

[66] Tran NQV, Miyake K (2017). Neurodevelopmental Disorders and Environmental Toxicants: Epigenetics as an Underlying Mechanism. Int J Genomics.

[67] Tabish AM, Poels K, Byun HM, Luyts K, Baccarelli AA, Martens J, Kerkhofs S, Seys S, Hoet P, Godderis L (2017). Changes in DNA Methylation in Mouse Lungs after a Single Intra-Tracheal Administration of Nanomaterials. PloS one.

[68] Oner D, Moisse M, Ghosh M, Duca RC, Poels K, Luyts K, Putzeys E, Cokic SM, Van Landuyt K, Vanoirbeek J (2017). Epigenetic effects of carbon nanotubes in human monocytic cells. Mutagenesis.

[69] Gorrochategui E, Li J, Fullwood NJ, Ying GG, Tian M, Cui L, Shen H, Lacorte S, Tauler R, Martin FL (2017). Diet-sourced carbon-based nanoparticles induce lipid alterations in tissues of zebrafish (Danio rerio) with genomic hypermethylation changes in brain. Mutagenesis.

[70] Ho SM (2010). Environmental epigenetics of asthma: an update. J Allergy Clin Immunol.

[71] Yadav S, Singh M, Yadav R. Organophosphates Induced Alzheimer’s Disease: An Epigenetic Aspect. J Clin Epigenet. 2016;2

[72] Wang A, Cockburn M, Ly T, Bronstein J, Ritz B. The association between ambient exposure to organophosphates and Parkinson's disease risk. Occup Environ Med. 2014. 10.1136/oemed-2013-101394 PMC4351788 24436061

[73] Fenech M (2007). Cytokinesis-block micronucleus cytome assay. Nat Protoc.

[74] OECD Guideline for the Testing of Chemicals. In Vitro Mammalian Cell Micronucleus Test (TG 487). 2016.

[75] OECD Guideline for the Testing of Chemicals. Mammalian Erythrocyte Micronucleus Test (TG 474). 2016.

[76] IAEA (2011). Cytogenetic Dosimetry: Applications in Preparedness for and Response to Radiation Emergencies, in: EPR-Biodosimetry 2011.

[77] Special Issue In vivo chemical Genotoxin Exposure and DNA damage in Humans measured using the lymphocyte Cytokinesis-Block Micronucleus Assay Mutat Research Review 2016, 770:1-2016.

[78] Nersesyan A, Fenech M, Bolognesi C, Misik M, Setayesh T, Wultsch G, Bonassi S, Thomas P, Knasmuller S (2016). Use of the lymphocyte cytokinesis-block micronucleus assay in occupational biomonitoring of genome damage caused by in vivo exposure to chemical genotoxins: Past, present and future. Mutat Res.

[79] Thomas P, Holland N, Bolognesi C, Kirsch-Volders M, Bonassi S, Zeiger E, Knasmueller S, Fenech M (2009). Buccal micronucleus cytome assay. Nat Protoc.

[80] Bolognesi C, Knasmueller S, Nersesyan A, Thomas P, Fenech M (2013). The HUMNxl scoring criteria for different cell types and nuclear anomalies in the buccal micronucleus cytome assay - an update and expanded photogallery. Mutat Res.

[81] Bolognesi C, Roggieri P, Ropolo M, Thomas P, Hor M, Fenech M, Nersesyan A, Knasmueller S (2015). Buccal micronucleus cytome assay: results of an intra- and inter-laboratory scoring comparison. Mutagenesis.

[82] Bolognesi C, Knasmueller S, Nersesyan A, Roggieri P, Ceppi M, Bruzzone M, Blaszczyk E, Mielzynska-Svach D, Milic M, Bonassi S, et al. Inter-laboratory consistency and variability in the buccal micronucleus cytome assay depends on biomarker scored and laboratory experience: results from the HUMNxl international inter-laboratory scoring exercise. Mutagenesis. 2016. 10.1093/mutage/gew047 27671865

[83] Holland N, Bolognesi C, Kirsch-Volders M, Bonassi S, Zeiger E, Knasmueller S, Fenech M (2008). The micronucleus assay in human buccal cells as a tool for biomonitoring DNA damage: the HUMN project perspective on current status and knowledge gaps. Mutat Res.

[84] Bolognesi C, Bonassi S, Knasmueller S, Fenech M, Bruzzone M, Lando C, Ceppi M (2015). Clinical application of micronucleus test in exfoliated buccal cells: A systematic review and metanalysis. Mutat Res Rev Mutat Res.

[85] Scheepers PB, Bos PM, Konings J, Janssen NAH, Grievink L (2011). Application of biological monitoring for exposure assessment following chemical incidents. A procedure for decision-making. J Expo Sci Environ Epidemiol.

[86] Manno M, Viau C, Cocker J, Colosio C, Lowry L, Mutti A, Nordberg M, Wang S, in-collaboration-with (2010). Biomonitoring for occupational health risk assessment (BOHRA). Toxicol Lett.

[87] Scheepers PTJ, Goën T (2017). Diagnosis.

[88] Edelman P, Osterloh J, Pirkle J, Caudill SP, Grainger J, Jones R, Blount B, Calafat A, Turner W, Feldman D (2003). Biomonitoring of chemical exposure among New York City firefighters responding to the World Trade Center fire and collapse. Environmental health perspectives.

[89] Boerleider R, Roeleveld N, Scheepers P (2017). Human biological monitoring of mercury for exposure assessment. AIMS Environmental Science.

[90] Bae YS, Kang SW, Seo MS, Baines IC, Tekle E, Chock PB, Rhee SG (1997). Epidermal growth factor (EGF)-induced generation of hydrogen peroxide. Role in EGF receptor-mediated tyrosine phosphorylation. J Biol Chem.

[91] Haddad JJ, Land SC (2002). Redox signaling-mediated regulation of lipopolysaccharide-induced proinflammatory cytokine biosynthesis in alveolar epithelial cells. Antioxid Redox Signal.

[92] Hsu HY, Wen MH (2002). Lipopolysaccharide-mediated reactive oxygen species and signal transduction in the regulation of interleukin-1 gene expression. J Biol Chem.

[93] Colavitti R, Pani G, Bedogni B, Anzevino R, Borrello S, Waltenberger J, Galeotti T (2002). Reactive oxygen species as downstream mediators of angiogenic signaling by vascular endothelial growth factor receptor-2/KDR. J Biol Chem.

[94] Junn E, Lee KN, Ju HR, Han SH, Im JY, Kang HS, Lee TH, Bae YS, Ha KS, Lee ZW (2000). Requirement of hydrogen peroxide generation in TGF-beta 1 signal transduction in human lung fibroblast cells: involvement of hydrogen peroxide and Ca2+ in TGF-beta 1-induced IL-6 expression. J Immunol.

[95] Ovrevik J, Refsnes M, Lag M, Holme JA, Schwarze PE (2015). Activation of Proinflammatory Responses in Cells of the Airway Mucosa by Particulate Matter: Oxidant- and Non-Oxidant-Mediated Triggering Mechanisms. Biomolecules.

[96] Thannickal VJ, Fanburg BL (2000). Reactive oxygen species in cell signaling. Am J Physiol Lung Cell Mol Physiol.

[97] Esposito F, Ammendola R, Faraonio R, Russo T, Cimino F (2004). Redox control of signal transduction, gene expression and cellular senescence. Neurochem Res.

[98] Schieber M, Chandel NS (2014). ROS function in redox signaling and oxidative stress. Curr Biol.

[99] Sauer H, Wartenberg M, Hescheler J (2001). Reactive oxygen species as intracellular messengers during cell growth and differentiation. Cell Physiol Biochem.

[100] Fisher AB (2009). Redox signaling across cell membranes. Antioxid Redox Signal.

[101] Barrett EG, Johnston C, Oberdorster G, Finkelstein JN (1999). Silica-induced chemokine expression in alveolar type II cells is mediated by TNF-alpha-induced oxidant stress. Am J Physiol Lung Cell Mol Physiol.

[102] Totlandsdal AI, Refsnes M, Lag M (2010). Mechanisms involved in ultrafine carbon black-induced release of IL-6 from primary rat epithelial lung cells. Toxicol In Vitro.

[103] Gabelloni ML, Sabbione F, Jancic C, Fuxman Bass J, Keitelman I, Iula L, Oleastro M, Geffner JR, Trevani AS (2013). NADPH oxidase derived reactive oxygen species are involved in human neutrophil IL-1beta secretion but not in inflammasome activation. Eur J Immunol.

[104] Trifilieff A, Walker C, Keller T, Kottirsch G, Neumann U (2002). Pharmacological profile of PKF242-484 and PKF241-466, novel dual inhibitors of TNF-alpha converting enzyme and matrix metalloproteinases, in models of airway inflammation. Br J Pharmacol.

[105] Deshpande SS, Angkeow P, Huang J, Ozaki M, Irani K (2000). Rac1 inhibits TNF-alpha-induced endothelial cell apoptosis: dual regulation by reactive oxygen species. Faseb J.

[106] Murphy C, Stack E, Krivelo S, McPartlin DA, Byrne B, Greef C, Lochhead MJ, Husar G, Devlin S, Elliott CT, O’Kennedy RJ (2015). Detection of the cyanobacterial toxin, microcystin-LR, using a novel recombinant antibody-based optical-planar waveguide platform. Biosensors and Bioelectronics.

[107] Crawley AS, O’Kennedy RJ (2015). The need for effective pancreatic cancer detection and management: a biomarker-based strategy. Expert Review of Molecular Diagnostics.

[108] Ma H, O’Kennedy RJ (2015). The Purification of Natural and Recombinant Peptide Antibodies by Affinity Chromatographic Strategies. Peptide Antibodies-series Methods in Molecular Biology.

[109] Loftus JH, Kijanka GS, O’Kennedy R, Loscher CE (2016). Patulin, deoxynivalenol, zearalenone and T-2 toxin affect viability and modulate cytokine secretion in J774A.1 murine macrophages. International Journal of Chemistry.

[110] Gilmartin N, Gião MS, Keevil CW, O'Kennedy RJ (2016). Differential internalin A levels in biofilms of Listeria monocytogenes grown on different surfaces and nutrient conditions. International Journal of Food Microbiology.

[111] Ayyar V, Sushrut A, O’Kennedy R (2016). Coming-of-Age of Antibodies in Cancer Therapeutics. Trends in Pharmacological Sciences.

[112] O'Reilly JA, Fitzgerald J, Fitzgerald S, Kenny D, Kay EW, O'Kennedy R, Kijanka GS (2015). Diagnostic potential of zinc finger protein-specific autoantibodies and associated linear B-cell epitopes in colorectal cancer. PloS one.

[113] Li H, Hedmer M, Kåredal M, Björk J, Stockfelt L, Tinnerberg H, Albin M, Broberg K (2015). A cross-sectional study of the cardiovascular effects of welding fumes. PloS one.

[114] Xu Y, Li H, Hedmer M, Hossain MB, Tinnerberg H, Broberg K, Albin M (2017). Occupational exposure to particles and mitochondrial DNA - relevance for blood pressure. Environmental Health.

[115] Li H, Hedmer M, Wojdacz T, Hossain MB, Lindh CH, Tinnerberg H, Albin M, Broberg K (2015). Oxidative stress, telomere shortening, and DNA methylation in relation to low-to-moderate occupational exposure to welding fumes. Environment Molecular Mutagenesis.

[116] Hossain MB, Li H, Hedmer M, Tinnerberg H, Albin M, Broberg K (2015). Exposure to welding fumes is associated with hypomethylation of the F2RL3 gene: a cardiovascular disease marker. Occupational Environmental Medicine.

[117] Alhamdow A, Gustavsson P, Rylander L, Jakobsson K, Tinnerberg H, Broberg K (2017). Chimney sweeps in Sweden: a questionnaire-based assessment of long-term changes in work conditions, and current eye and airway symptoms. International Archives of Occupational Environmental Health.

[118] Marie-Desvergne C, Maître A, Bouchard M, Ravanat JL, Viau C (2010). Evaluation of DNA adducts, DNA and RNA oxidative lesions, and 3-hydroxybenzo(a)pyrene as biomarkers of DNA damage in lung following intravenous injection of the parent compound in rats. Chem Res Toxicol.

[119] Brown TP, Rumsby PC, Capleton AC, Rushton L, Levy LS (2006). Pesticides and Parkinson's disease--is there a link?. Environmental health perspectives.

[120] Baldi I, Cantagrel A, Lebailly P, Tison F, Dubroca B, Chrysostome V, Dartigues JF, Brochard P (2003). Association between Parkinson's disease and exposure to pesticides in southwestern France. Neuroepidemiology.

[121] Baldi I, Lebailly P, Mohammed-Brahim B, Letenneur L, Dartigues JF, Brochard P (2003). Neurodegenerative diseases and exposure to pesticides in the elderly. Am J Epidemiol.

[122] Liew Z, Wang A, Bronstein J, Ritz B (2014). Job exposure matrix (JEM)-derived estimates of lifetime occupational pesticide exposure and the risk of Parkinson's disease. Arch Environ Occup Health.

[123] Elbaz A, Clavel J, Rathouz PJ, Moisan F, Galanaud JP, Delemotte B, Alperovitch A, Tzourio C (2009). Professional exposure to pesticides and Parkinson disease. Ann Neurol.

[124] Feldman AL, Johansson AL, Nise G, Gatz M, Pedersen NL, Wirdefeldt K (2011). Occupational exposure in parkinsonian disorders: a 43-year prospective cohort study in men. Parkinsonism Relat Disord.

[125] van der Mark M, Vermeulen R, Nijssen PC, Mulleners WM, Sas AM, van Laar T, Brouwer M, Huss A, Kromhout H (2014). Occupational exposure to pesticides and endotoxin and Parkinson disease in the Netherlands. Occup Environ Med.

[126] Kamel F, Tanner C, Umbach D, Hoppin J, Alavanja M, Blair A, Comyns K, Goldman S, Korell M, Langston J (2007). Pesticide exposure and self-reported Parkinson's disease in the agricultural health study. Am J Epidemiol.

[127] Ritz BR, Paul KC, Bronstein JM (2016). Of Pesticides and Men: a California Story of Genes and Environment in Parkinson's Disease. Curr Environ Health Rep.

[128] Goldberg DW, Wilson JP, Knoblock CA, Ritz B, Cockburn MG (2008). An effective and efficient approach for manually improving geocoded data. Int J Health Geogr.

[129] Costello S, Cockburn M, Bronstein J, Zhang X, Ritz B (2009). Parkinson's disease and residential exposure to maneb and paraquat from agricultural applications in the central valley of California. Am J Epidemiol.

[130] Wang A, Costello S, Cockburn M, Zhang X, Bronstein J, Ritz B (2011). Parkinson's disease risk from ambient exposure to pesticides. Eur J Epidemiol.

[131] Narayan S, Liew Z, Paul K, Lee PC, Sinsheimer JS, Bronstein JM, Ritz B (2013). Household organophosphorus pesticide use and Parkinson's disease. Int J Epidemiol.

[132] Gatto NM, Cockburn M, Bronstein J, Manthripragada AD, Ritz B (2009). Well-water consumption and Parkinson's disease in rural California. Environmental health perspectives.

[133] Fitzmaurice AG, S. L., Rhodes SL, Lulla A, Murphy NP, Lam HA, O’Donnell KC, Barnhill L, Casida JE, Cockburn M, Sagasti A (2013). Aldehyde dehydrogenase inhibition as a pathogenic mechanism in Parkinson disease. Proc Natl Acad Sci U S A.

[134] Paul KC, Sinsheimer JS, Rhodes SL, Cockburn M, Bronstein J, Ritz B (2016). Organophosphate Pesticide Exposures, Nitric Oxide Synthase Gene Variants, and Gene-Pesticide Interactions in a Case-Control Study of Parkinson's Disease, California (USA). Environmental health perspectives.

[135] Rhodes SL, Fitzmaurice AG, Cockburn M, Bronstein JM, Sinsheimer JS, Ritz B (2013). Pesticides that inhibit the ubiquitin-proteasome system: effect measure modification by genetic variation in SKP1 in Parkinsons disease. Environ Res.

[136] Kannarkat GT, Cook DA, Lee JK, Chang J, Chung J, Sandy E, Paul KC, Ritz B, Bronstein J, Factor SA, et al. Common Genetic Variant Association with Altered HLA Expression, Synergy with Pyrethroid Exposure, and Risk for Parkinson's Disease: An Observational and Case-Control Study. NPJ Parkinsons Dis. 2015;1 10.1038/npjparkd.2015.2 PMC4853162 27148593

[137] Lee PC, Rhodes SL, Sinsheimer JS, Bronstein J, Ritz B (2013). Functional paraoxonase 1 variants modify the risk of Parkinson's disease due to organophosphate exposure. Environ Int.

[138] Proceedings of the 2nd International DiMoPEx Conference on “Pollution in living and working environments and health”, DiMoPEx Working Groups Meeting. Journal of Health and Pollution 2017, 8:in press.

[139] Balbus JM, Barouki R, Birnbaum LS, Etzel RA, Gluckman PD, Grandjean P, Hancock C, Hanson MA, Heindel JJ, Hoffman K (2013). Early-life prevention of non-communicable diseases. Lancet.

[140] Bloom D, Cafiero E, Jané-Llopis E, Abrahams-Gessel S, Bloom L, Fathima S, Feigl A, Gaziano T, Mowafi M, Pandya A (2011). The Global Economic Burden of Noncommunicable Diseases.

[141] Environment and Human Health (2013). Joint EEA-JRC report. EEA Report No 5/2013.

[142] Norman RE, Carpenter DO, Scott J, Brune MN, Sly PD (2013). Environmental exposures: an underrecognized contribution to noncommunicable diseases. Rev Environ Health.

[143] Prüss-Üstün A, Wolf J, Corvalán C, Bos R, Neira M (2016). Preventing disease through healthy environments A global assessment of the burden of disease from environmental risks.

[144] Vineis P, Stringhini S, Porta M (2014). The environmental roots of non-communicable diseases (NCDs) and the epigenetic impacts of globalization. Environ Res.

[145] WHO (2016). Ambient air pollution: a global assessment of exposure and burden of disease.

[146] WHO (2016). The public health impacts of chemicals: knowns and unknowns.

[147] WHO Regional Office for Europe (2013). Health 2020: a European policy framework supporting action across government and society for health and well-being.

[148] WHO Regional Office for Europe (2015). The Minsk Declaration – The Life-course Approach in the Context of Health 2020.

[149] WHO Regional Office for Europe (2015). Strategic Approach to International Chemicals Management: implementation and priorities in the health sector. Meeting report.

[150] WHO Regional Office for Europe. Sixth Ministerial Conference on Environment and Health, Copenhagen. http://www.eurowhoint/en/media-centre/events/events/2017/06/sixth-ministerial-conference-on-environment-and-health/read-more 2017a.

[151] WHO Regional Office for Europe. Sustainable Development Goals, Copenhagen http://www.eurowhoint/en/health-topics/health-policy/sustainable-development-goals-sdgs 2017b. 28956895

[152] WHO Regional Office for Europe. Chemical policies and programmes to protect human health and environment in a sustainability perspective. Meeting report, Copenhagen. http://www.eurowhoint/__data/assets/pdf_file/0009/334665/Chemical-safety-meeting-report_new-coverpdf 2017c.

[153] WHO Regional Office for Europe. Implementation of the Minamata Convention in the health sector: challenges and opportunities, Copenhagen. http://www.eurowhoint/en/health-topics/environment-and-health/health-impact-assessment/publications/2017/implementation-of-the-minamata-convention-in-the-health-sector-challenges-and-opportunities-2017 2017d.

