# Supplementary material for: Diagnosis, monitoring and prevention of exposure-related non-communicable diseases in the living and working environment: DiMoPEx-project is designed to determine the impacts of environmental exposure on human health
Source: J Occup Med Toxicol. 2018 Feb 5;13:6. doi: 10.1186/s12995-018-0186-9 (PMC5800006; doi:10.1186/s12995-018-0186-9)
Supplement: Additional file 1: — Info Box 1–-3. Tables 1–-11. Including additional references ([139–153]). (DOCX 504 kb) [file 12995_2018_186_MOESM1_ESM.docx]

**ADDITIONAL FILE 1**

| Info Box 1  Environmental and occupational factors in the burden of NCDs |
| --- |
| NCDs – mainly CVDs, cancers, chronic respiratory diseases, and diabetes – account for 63% of global deaths [[1](#_ENREF_1)], and are the leading cause of disability globally, with substantial associated economic burden, projected to increase in the coming decades [[2](#_ENREF_2)].  Virtually all chronic diseases are the outcome of an interplay of genetic, host, and environmental factors. Whereas the understanding of multiple causal pathways and interactions remains limited, the role of occupational and environmental factors is increasingly recognized [[3](#_ENREF_3)][[4](#_ENREF_4)][[5](#_ENREF_5)][[6](#_ENREF_6)].  According to WHO [[7](#_ENREF_7)], modifiable environmental factors are responsible for 23% of global deaths and 26% of deaths in children aged under 5 years. Out of the total 12.6 million deaths attributable to unhealthy environments, NCDs represent a majority (8.2 million); the leading causes are stroke, ischemic heart disease, followed by cancer and chronic respiratory diseases [[7](#_ENREF_7)]. The fraction of NCDs in the global burden of diseases attributable to the environment is growing, with some regional differences. While the overall environment-related disease burden is much higher in lower-income countries, for NCDs such as CVDs and cancers, the per capita disease burden is greater in the developed world. Some countries experience a “double burden” of unresolved issues of communicable diseases and a growing burden of NCDs, coinciding with rapid lifestyle and environmental changes [[7](#_ENREF_7)]. The burden of disease linked to modifiable environmental factors also indicates a potential for public health gains through prevention, focused on reducing environmental risk factors and supporting high-quality environments; this requires efforts well beyond the health sector, involving a broad range of policies, strategies, interventions, technologies, and knowledge.  In the WHO European Region, the new health policy framework, Health 2020, aims to “significantly improve the health and well-being of populations, reduce health inequalities, strengthen public health and ensure people-centred health systems that are universal, equitable, sustainable and of high quality” [[1](#_ENREF_1)]. The four mutually supportive priority areas – a life-course approach to health, tackling major burdens of NCDs and communicable diseases, strengthening health systems, and creating supportive environments and resilient communities – provide a robust framework for regional efforts. Aligned with this policy is the European Environment and Health Process, which recently set regional priorities for action in environment and health at the Sixth Ministerial Conference in Ostrava, Czechia, in June 2017 [[8](#_ENREF_8)]. The regional efforts contribute to the implementation of the 2030 Agenda on Sustainable Development and its goals, focusing on health [[7](#_ENREF_7)][[9](#_ENREF_9)].  Air pollution is the biggest environmental risk to health, responsible for the premature deaths of 6.5 million people every year globally and more than 600 000 in the WHO European Region. Worldwide (based on 2012 data), the most common causes of premature death attributable to ambient (outdoor) air pollution are ischemic heart disease and stroke (72%), followed by chronic obstructive pulmonary disease, lung cancer, and other respiratory diseases, including asthma [[10](#_ENREF_10)]. Therefore, improving air quality can deliver substantial health benefits in terms of reducing premature deaths and diseases. In 2016, WHO released the updated database on ambient and household air pollution (with concentrations of particulate matter in over 3000 cities in 103 countries worldwide), and the estimates of the burden of disease from ambient and household air pollution worldwide, and by region [[10](#_ENREF_10)].  Hazardous chemicals in air, water, food, consumer products and occupational environment have the potential of contributing to the burden of NCDs and disorders. While concerns about public health impacts of exposure to chemicals are growing, abilities to assess and quantify them are limited. In 2016, WHO assessed public health impacts of a few well-known chemical hazards, for which the data and the evidence to assess the burden of diseases were available. These included, for example, lead, occupational carcinogens, second-hand smoke, and chemicals involved in acute poisonings, but not chemicals such as endocrine-disrupting chemicals, which represent a big and heterogenous group of compounds. Around 1.3 million deaths were attributable to those few chemical exposures; exposure to lead alone accounts for 667 000 deaths annually, and unintentional poisonings are estimated to cause 193 000 deaths annually [[11](#_ENREF_11)].  Not captured in these estimates are exposures in early childhood, while the evidence is growing on links between early-life exposures to chemicals and several NCDs later in life. In the Ministerial Declaration from the Conference on The Life-course Approach in the Context of Health 2020 “investment in early childhood development and protection against toxic stress and dangerous environmental exposure at critical points of development” has been identified as among “the most cost-effective policy choices” available to governments [[12](#_ENREF_12)].  The challenge of chemical exposures and human health requires multidirectional action. In the WHO European Region, efforts are undertaken to identify regional priorities for sound management of chemicals with health sector involvement [[13](#_ENREF_13)], to build capacity to address risks of hazardous chemicals, advocate and advise on the development of chemical policies [[14](#_ENREF_14)], support implementation of multilateral agreements, such as the Minamata Convention, focusing on the role of health sector [[15](#_ENREF_15)], and provide technical assistance in preparedness and response to chemical emergencies. |
|   *Source*: Prüss-Üstün et al. [[7](#_ENREF_7)]. |

| Info Box 2  New insights into toxicology: an integrated experimental approach for hazard identification |
| --- |
| For nearly five decades long-term studies in rodents have been the accepted benchmark for assessing chronic long-term toxic effects, particularly carcinogenicity, of chemicals. The European Food Safety Authority (EFSA) and the World Health Organization (WHO) have pointed out that the current set of internationally utilized test methods capture only some of the potential adverse effects associated with lifetime exposures to these agents over the lifetime. Current OECD guidelines (see ref. [[16](#_ENREF_16)]) as planned, are not aimed at monitoring cancer hazards and risks of exposure on susceptible individuals such as children and the elderly. Under current testing procedures, relatively high doses of a chemical are given to animals, generally higher than the doses to which humans are exposed. When conducting cancer bioassays, it is therefore important to investigate the effect of low doses (sometimes many orders of magnitude lower than the No Observed Adverse Effect Level _NOAEL in animals) and a systematic dose-calibration study should be performed in an appropriate rodent model in order to identify the relevant administered oral dose of the test substance that results in biomarker concentrations (e.g. urine, serum) comparable to those ones observed in human population (see ref. [[17](#_ENREF_17)]). Furthermore, based on the results of long-term carcinogenicity bioassays used for testing chemical and physical agents on using rodents, there is ample evidence demonstrating that exposures during early developmental phases, produce an overall increase of malignant tumors and increases of specific organ site neoplasms related to exposures to specific carcinogens, such as in the case of vinyl chloride and benzene. Use of lifelong and early exposure models provides a greater opportunity to construct comprehensive mechanistic models of disease. |

| Table 1  Fact sheet: A human-equivalent animal model in rodent bioassay for primary prevention | |
| --- | --- |
| Animal model | **Characteristics of Carcinogenicity studies** |
| Sprague-Dawley (SD) rats are the strain used at the Ramazzini Institute (RI)* and the National Toxicology Program, on the basis of the evidence that they are adequately sensitive, have a long history of being used in research studies, and are also recommended by the Organization for Economic Cooperation and Development (OECD). | **A distinctive characteristic of most RI carcinogenicity studies is that exposure starts from prenatal life and rodents are kept under observation until natural death or at least 130 weeks of age. This lifespan protocol is in contrast with most laboratories where rodents are often treated starting from 8 weeks of age and sacrificed at 110 weeks of age (representing about 2/3 of the lifespan) and corresponding to 60-65 years in humans. The Ramazzini Institute aims therefore to investigate a “human equivalent model”. In fact, in humans the exposure to toxic agents, even at very low doses, starts during gestation, continues through lactation (mother’s milk) and lasts until spontaneous death.** |
| *RI (The Ramazzini Institute) is an independent, non-profit cooperative, which manages the Cesare Maltoni Cancer Research Center (CMCRC) in Italy, where one of the world’s largest and longest existing programs of carcinogenicity bioassays is conducted, second only to that of the United States’ National Toxicology Program (NTP). The Ramazzini methodology is characterized by systematic and integrated project design, consistency over time, and homogeneity of approach. Agents are selected for study on the basis of their potential diffusion and on the adequacy of existing experimental evidence. | |

**Info Box 3**

**Exposure related cancer: simplified mode of action and some examples of cancer causing agents/processes (IRAC)**

**Table 2 Exposure related cancer**

| Cancer target organ | Agents/processes to be carcinogen for human |
| --- | --- |
| lung cancer (lung and bronchus) | **asbestos; talc containing asbestiform fibres; christaline silica; arsenic/arsenic compounds; hexavalent chromium/chromium compounds; nickel; cobalt; beryllium/ beryllium compounds; inorganic lead; PAHs (**[**polycyclic aromatic hydrocarbons**](https://en.wikipedia.org/wiki/Polycyclic_aromatic_hydrocarbon)); **radon/ionizing radiation; mustard gas; underground hematic mining; iron and steel founding (chromium, silica, PAHs, phenol, isocyanates, amines, formaldehyde mixtures); tin miners; coals gasification; coke production/ coke oven gas; soot; strong inorganic mist (containing sulfuric acid); welding; bis (chlormethylether, chlormethyl ether; paint manufacturing/ spray painting; TCDD (2,3,7,8-terachlordibenzene dioxin); diesel engine exhaust; mineral oil;**  **tobacco smoke (50 known carcinogens inkl. nitrosamines and** [**polycyclic aromatic hydrocarbons**](https://en.wikipedia.org/wiki/Polycyclic_aromatic_hydrocarbon)**); ETS (non-smokers)** |
| tumors of hemato-poietic and lymphoid tissues (i.e. multiple myeloma , ALL; CLL; AML, CML, NHL AMoL) | **benzene, ionizing radiation, pesticides (particularly: organophosphates, DDT, heptachlor, chlordane phenoxi acid herbicides, phosphining acids, HCB, lindane, oxychlordane, lindane, TCDD, fungicide captafol); pesticide applicators , especially chlorinated insecticide dieldrin and fumigant mixtures (carbon tetrachloride + carbon disulfide aromatic hydrocarbons, formaldehyde** |
| urothelium (i.e. urinary tract, bladder cancer) | **aromatic amines, halogenated alkyl-/aryl-oxides,** |
| naso-pharyngeal cancer | **chrom VI, nickel, arsenic, formaldehyde, organic solvents,**  **wood dust, welding fumes, farming, construction, leather dust,** |
| larynx cancer | [**polycyclic aromatic hydrocarbons**](https://en.wikipedia.org/wiki/Polycyclic_aromatic_hydrocarbon) |
| mesotheliom/pleural lining of the lungs, heart, or abdomen | **asbestos, pitch tar bitumen** |
| skin cancer/ melanom | **UV light; ionizing radiation,** [**polycyclic aromatic hydrocarbons**](https://en.wikipedia.org/wiki/Polycyclic_aromatic_hydrocarbon) |

| Table 3  Agents are selected for study on the basis of their potential diffusion and on the adequacy of existing experimental evidence* | | | |
| --- | --- | --- | --- |
| Agent | **Year** | **Before** | **After** |
| Vinyl choride | 1974 | Limit 500 ppm | Limit 1 ppm |
| Benzene | 1979 | Not classified | Carcinogenic (IARC 1) |
| Trichlorethylene | 1988 | Not classified | Carcinogenic (IARC 1) |
| Formaldehyde | 1989 | Not classified | Carcinogenic (IARC 1) |
| Asbestos | 1991 | Associated only to lung cancer and mesothelioma | Also associated with larynx, ovary, pharynx, stomach, colon and rectum cancer |
| Mancozeb | 2002 | Not classified | Carcinogenic (Prob 65) |
| Fluoro-Edenitic Fibres | 2004 | Not classified | Carcinogenic (IARC 1) |
| *At the CMCRC, studies have been conducted on more than 200 compounds present in the industrial and the general environment. Substances demonstrated to be carcinogenic include: vinyl chloride, benzene, formaldehyde, trichlorethylene, fuels and their components and additives, pesticides, and recently aspartame. The results obtained from most of these studies have formed the basis of rules and regulations for primary prevention, even if sometimes many years have passed over before having the confirmation of multipotent carcinogenicity. | | | |

| Table 4  Current two years experiments may mask a possible carcinogenic response | |
| --- | --- |
| Xylenes: | **Xylenes were administered to 40 male and 40 female Sprague-Dawley rats by stomach tube at concentrations of 500 or 0 mg/kg b.w. in extra virgin olive oil, once daily 4 or 5 days per week, for 104 weeks beginning at 7 weeks of age. The control group received olive oil alone. Animals were observed until spontaneous death occurred. An increase was observed in total malignant tumors, mammary and oral cavity carcinomas, and hemolymphoreticular neoplasias. The increase in total malignant tumors, oral cavity carcinomas, and hemolymphoreticular neoplasias was only observed after 112 weeks of age It should be noted that the experiment with xylene performed by the National Toxicology Programme (NTP) sacrificed rats after 104 weeks of treatment; no carcinogenic effect was found** |
| Mancozeb | **Mancozeb is a pesticide, ranked among the EU list of Endocrine Disruptive Chemicals (EDCs). It was administered to 75 male and 75 female Sprague-Dawley rats with feed at concentrations of 1,000, 500, 100, 10, or 0 ppm supplied ad libitum for 104 weeks beginning at 8 weeks of age. All animals were observed until spontaneous death. Control animals received standard feed. Among other findings, a strong increase in malignant tumors of the thyroid gland in males and females was observed after 112 weeks of age** |

| Table 5  Circulating nucleic acids as diagnostic and prognostic markers | |
| --- | --- |
| cfmiRNAs | **MiRNAs consist of 19 to 25 nucleotides, are single-stranded and derived from hairpin precursor molecules of 70-100 nucleotides. As one of the largest gene family miRNAs account for approx. 1% of the human genome and are highly conserved in nearly all organisms. In mammals, they are believed to regulate approx. 50% of all protein-coding genes. Mostly, they are post-transcriptional regulators that sequence-specifically bind to complementary sequences of the 3’-untranslational region (UTR) of their target mRNAs (and occasionally 5´UTR or coding sequences). In this way, the protein expression is post-transcriptionally repressed either by inhibiting the translation or degrading their target mRNA [**[**18**](#_ENREF_18)**]. In the blood, miRNAs circulate in a highly stable form and are protected against RNase digestion, because most of them are included in apoptotic bodies, microvesicles, exosomes and complexes with RNA-binding proteins [**[**19**](#_ENREF_19)**].** |
| Exosomes | **Exosomes are a major class of extracellular vesicles that are 40-100 nm in diameter. They are formed via the endolysosomal pathway. These small membrane particles are actively released from multiple cell types, including dendritic cells, lymphocytes and tumor cells, by exocytosis [**[**20**](#_ENREF_20)**].They are involved in cell-to-cell communication by transferring their cargo of DNA, RNA, miRNAs and lipids between cells.** |

| Table 6  Fact sheet: Epigenetic regulations during lifespan | |
| --- | --- |
| Epigenetic regulation | **Epigenetic regulation occurs during the entire lifespan and involves four layers of tightly controlled mechanisms: DNA methylation, histone modifications, chromatin remodeling and microRNA expression. It is conceivable that a mechanisms precisely controlled like epigenetic, could be affected by external factors. Indeed, although epigenetic alterations are primarily linked to development and cancer, it is now becoming evident that the environment or, in general, external stimuli can influence the epigenetic asset and that the exposure to chemicals and carcinogens affects the epigenetic landscape. Importantly, epigenetic alterations and in particular DNA methylation, are regarded as early events of cell transformation including both global and gene-specific hypo methylation (causing genomic instability and inappropriate activation of proto-oncogenes) as well as gene-specific CpG island promoter hyper methylation (which induces silencing of cancer-related genes with tumor suppressive properties).** |

| Info Box 4  HUMN project provides standardized protocol for buccal cell collection |
| --- |
| A standardized protocol for buccal cell collection, slide preparation and scoring was established by the HUMN project consortium by taking into account the available confounding factors and staining artifacts [[21](#_ENREF_21)]. A comprehensive description of the scoring criteria to identify different cell types and nuclear anomalies accompanied by a photo gallery is also available [[22](#_ENREF_22)]. The results of inter-calibration studies revealed that the scoring criteria are sufficiently robust to obtain consistent results concerning the identification of differentiated cells and micronuclei, although a proper training by experienced laboratory scientists is mandatory [[23](#_ENREF_23), [24](#_ENREF_24)]).  The assay was widely applied in biomonitoring studies to evaluate occupational or environmental exposures to genotoxic agents, medical procedures, micronutrient deficiencies and lifestyle factors [[25](#_ENREF_25)]. The large majority of data on the MN assay in buccal cells concerns occupational and environmental exposures to genotoxic agents through inhalation or by direct contact. Significant increases of MN frequency were observed in buccal cells from people exposed to genotoxic compounds.  It was also suggested that MN test in buccal mucosa may predict cancer risk. The large majority of the available data on cancer patients are related to the upper aero digestive tract although a number of studies are available on patients with other tumors. |

| Table 7: Different types of biomarkers with some examples and how they can be interpreted and used in research. | | | |
| --- | --- | --- | --- |
| Class | **Example** | **Observation** | **Interpretation** |
| EXPOSURE | **Parent substance in blood** | **Systemic presence of a xenobiotic free in the circulation** | **Chemical marker reflecting absorption and systemic presence in terms of ‘body burden’** |
|  | **Metabolite urine** | **Biotransformation of the parent substance to a more or less active species** | **Chemical marker reflecting absorption and systemic bioavailability and including elimination** |
|  | **Protein adduct in blood cells** | **Covalent binding of a reactive intermediate as a post translational change to a protein primary structure** | **Chemical marker reflecting absorption and systemic presence, bioavailability and bio-activation** |
|  | **DNA-adduct in blood cells** | **Covalent binding of a reactive intermediate to a DNA-base** | **Chemical marker reflecting absorption and systemic presence, bioavailability, bio-activation and DNA repair** |
|  | **Parent substance in exhaled air** | **Concentration in equilibrium with the arterial blood concentration** | **Reflects the target dose of the brain and other internal organs** |
| RESPONSE | **Micronuclei, chromosome aberrations, sister chromatid exchanges** | **Lesions in chromatin and DNA conformation and structure** | **Indication of genotoxicity with potential health consequences if lesions occur in critical regions and are beyond repair** |
|  | **DNA-repair products** | **DNA repair products that reflect DNA-damage but do not provide structure information on the causing agent** | **Outcome of a number of events at a molecular level, related to both damage and repair** |
|  | **Oxidative damage** | **Oxidative damage in proteins, DNA and RNA.** | **Reflecting non-specific molecular damage by reactive oxygen and nitrogen species that was not removed by repair** |
|  | **GST genotype** | **Expression of constitutional factor e.g. in biotransformation of endogenous and exogenous chemicals** | **Presence of isoforms of enzymes involved in one or more metabolic steps e.g. leading to conjugation of electrophilic intermediates** |
|  | **CYP phenotype** | **CYP activity based on substrate-product conversions and metabolite ratios** | **Activity of (isoforms of) enzymes that changes the chemistry of a chemical agent to become more water soluble and more or less toxic** |
|  | **DNA methylation** | **Methylation of DNA bases cytosine and adenine** | **Gene modulation in carcinogenesis (hyper-methylation of tumor suppressor genes and hypo-methylation of oncogenes)** |

| Table 8: Examples of preventable problems during human biological monitoring campaigns. | | | | |
| --- | --- | --- | --- | --- |
|  | **Task** | **Potential pitfall** | **Example** | **Contingency plan** |
| SUTDY DESIGN | **Sample coding system** | **Poor annotation** | **Collecting spot urine samples without proper administration of urine void times** | **Instruction of study participants to keep a log of times of urine voiding** |
|  | **Biomarker selection** | **Poor decision** | **Use of a nonspecific marker such as hippuric acid to assess toluene exposure** | **Careful preparation, including well-informed decisions concerning choice of biomarkers** |
| ETHICS | **Preparation of study protocol** | **Privacy protection breach** | **Incomplete or improper data protection management plan** | **Careful preparation, including well-informed decisions concerning ethics requirements** |
|  | **Preparation of participant information** | **No clearance for use of biomaterials** | **Inaccurate description of study objective in participant information e.g. with reference to specification of endpoints** | **Careful description of type of endpoints and possible future use of biomaterials in the context of related research questions that require additional use of collected biomaterials. For toluene the parent substance or o-cresol are suitable alternative biomarkers** |
| INFOR-MATION | **Participant instruction** | **Loss of sample** | **Not collect information on bladder infection when collecting urine** | **Retrieve information on bladder infection, use rigorous cooling facilities/procedures and/or add chemical preservative to urine** |
|  |  | **Missed fist void** | **Participants not well instructed how to collect first morning void of urine** | **Instruct participant to place the collection container on the closed toilet seat and/or send a personal reminder to the phone of the participant.** |
| TECHNICAL | **Sample collection** | **Contamination** | **Use of stainless steel needles for blood collection in a trace metal study** | **Pre-testing of all materials and use silicone liner in needles to prevent contamination of the blood sample with metals.** |
|  |  | **Matrix problems** | **Blood sampling following a meal contains high fat interacting with sample analysis** | **Planning of time of sample collection** |
|  | **Sample shipment and storage**  **Biobanking** | **Degradation due to sterility breach** | **Urine samples were kept in transit at ambient temperature on an intl. airport** | **Change the pH of the sample by addition of acid directly after sample collection** |
|  |  | **Contamination during storage** | **Diffusion of solvent vapor on solid sorbent from exhaled air sample** | **Do not mix biomaterials storage with general laboratory storage** |
|  |  | **Degradation** | **Chemically unstable biomarker** | **Addition of internal standard before samples are stored** |
|  |  | **Sample code** | **Use of labels that fall of in a freezer** | **Use of high quality labels or use chips** |
| INTERPRETA-TION | **Participant instruction** | **Spot urine** | **Not able to calculate excretion rate due to lack of data on time lag between voids** | **Include a complete administration of void times and explain why it is important to have a complete time record of urine void times** |
|  | **Participant instruction** | **Co-exposures** | **Prevent obvious sources of co-exposures such as seafood consumption in study on arsenic or consumption of smoked/fried/ barbecued meat for PAH biomonitoring** | **Careful preparation, including well-informed decisions concerning potential interacting co-exposures** |

| Table 9  Reactive oxygen species (ROS) and oxidative stress in particle induced toxicity | |
| --- | --- |
| Particle induced ROS formation | To determine with certainty whether the effects of antioxidant treatments are due to interference with the particles’ oxidative capacity or direct particle-induced ROS formation, is however difficult. Endogenous redox-responses are also involved in the regulation of a variety of complex cellular processes such as mitogenic signal transduction, phagocytosis, gene expression, regulation of cell proliferation, replicative senescence and apoptosis, and are essential effectors in the inflammatory response. If this central, endogenous role of red-ox regulation in cellular processes is overlooked, one might end up attributing effects of antioxidants to interference with the oxidative capacity of particles and exogenous oxidative stress, also in cases when an equally or perhaps more likely explanation is that the antioxidants interfered with normal signaling pathways or cell-generated oxidative stress. By comparison, a variety of other (non-particulate) endogenous and exogenous agents have been reported to induce ROS-generation as part of their effects on cells, including endotoxin, growth factors, cytokines and extracellular calcium, which therefore can be attenuated by antioxidants. |
| ROS production | ROS tend to be produced inside vesicles and organelles such as endosomes, lysosomes and the endoplasmic reticulum (ER), or extracellular outside the plasma membrane, and not freely in the cytoplasm. H_2_O_2_ may then be channeled through the membrane through aquaporins, while O_2_^●-^ appears to pass through anion channels or could be transformed to H_2_O_2_ by SOD in the extracellular or vesicular compartments.. |

| Table 10. Endogenous redox-regulation.  Reactive oxygen species (ROS) are generated by a variety of cellular enzymes and through the mitochondrial respiratory chain. To avoid damage to cellular constituents, ROS production is tightly regulated and excess ROS is rapidly inactivated by cellular antioxidants. | | |
| --- | --- | --- |
| Cellular ROS production | **Antioxidant defence** | |
|  | *Enzymatic* | *Non-enzymatic* |
| Cyclooxygenases  Cytochrome P450  γ-Glutamyl transpeptidase  Lipoxygenases  NADPH oxidase  Mitochondria  Xanthine oxidases | Catalase  Glutathione peroxidase  Peroxiredoxins  Prion protein (PrP^c^)  Superoxide dismutases | Glutaredoxin  Glutathione  Lipoate  Pyruvate  Thioredoxin  Ubiquinone  Urate  Vitamin A, C and E |

| Table 11. Projects to generate markers that sign human exposures within DiMoPEx antibody center | |
| --- | --- |
| Assay outputs | **Assays already generated** |
|  | **Biomarkers of prostate, colorectal and pancreatic cancer** |
|  | **Markers of cardiac disease: myeloperoxidase, fatty acid binding protein and troponins** |
|  | **Food contaminants e.g. aflatoxins** |
|  | **Environmental toxins e.g. microcystins** |
|  | **Fully commercialized assay for CRP detection** |
|  | **Pathogens e.g. Listeria** |

| Info Box 5  HUMAN DATA: The general data protection regulation |
| --- |
| New kinds of data are rapidly becoming available in massive quantities, providing a record of the transactions we carry out, the communications we make and other social and economic activities. Although such data are not collected primarily for research, they offer considerable research potential and have the capacity to yield improved insights into society, health, the economy and political behavior. However, along with the potential benefits, the availability of these data in a rapidly changing digital environment also presents a number of ethical challenges. These include risks relating to the disclosure of the identities of individuals or organisations; reputational risks for organizations collecting or creating data; and issues around the ethics of research using these data.  In 2016, the OECD published a policy paper compiled by an expert group, appointed by the OECD Global Science Forum (GSF), that brought together a wide range of expertise from many countries. The Group worked to address what is rapidly becoming one of the most pressing challenges for scientists from multiple disciplines– research use of new forms of data. Their report examined more than question of the legality of such usage, but of ‘doing the right thing’. (OECD (2016), “Research Ethics and New Forms of Data for Social and Economic Research”, OECD Science, Technology and Industry Policy Papers, No. 34, OECD)  The General Data Protection Regulation (GDPR) is a new piece of data protection regulation which will become law across the EU in May 2018. It will replace all current data protection regulations within member jurisdictions and has wider-anging implications for protection of personal data and identifiers. The GDPR introduces some new definitions of certain special categories of personal data whose processing is explicitly forbidden, by principle, but may be admitted for research or archiving purposes in exceptional circumstances in the public interest in the respect of Articles 9 and 89 of the GDPR. It further provides definitions of data concerning health and genetic data which will impact on the practical implementation of some research. The GDPR establishes a new system using a risk-based approach. This new approach will be implemented through an integrated system of data protection, and will be reliant upon new procedures which will be applied in scientific research settings.  It is intended that the GDPR will preserve the equilibrium between the necessity of effectively protecting data subjects’ rights in a digitalised and globalised world while allowing the processing of personal data, including sensitive data, for scientific research. It reinforces cooperation duties and transparency between the actors of the processing, internally and with regard to the supervisory authorities, which should create a more integrated EU data protection system and diminish some useless administrative costs by decentralising elements of the data protection governance towards data controllers and processors [[26](#_ENREF_26)]. |

| Table 12. Ethical reference framework, Research Ethics Committees and Data protection framework under EU law | |
| --- | --- |
| Ethical framework | World Medical Association [*Declaration of Helsinki 2000*](http://www.wma.net/e/policy/b3.htm)  World Health Organization,  [*Guidelines for Good Clinical Practice for Trials on Pharmaceutical Products (1995)*](http://www.vghtpe.gov.tw/~mre/goodexp/Fercap-Survey/WHO-GCP-1995.pdf)  International Conference on Harmonisation of Technical Requirements for Registration of Pharmaceuticals for Human Use (ICH) [*Guidelines for Good Clinical Practice (1996)*](http://www.ich.org/cache/compo/276-254-1.html)  “Oviedo Convention” - [*Council of Europe Convention on Human Rights and Biomedicine*](http://conventions.coe.int/Treaty/Commun/QueVoulezVous.asp?NT=164&CL=ENG) and it's additional protocols, particularly the Additional Protocol concerning Biomedical Research (January 2005)  CIOMS (Council for International Organizations of Medical Sciences) [*International Ethical Guidelines for Biomedical Research Involving Human Subjects (2002)*](http://www.cioms.ch/guidelines_nov_2002_blurb.htm)  [Universal Declaration of Human Rights (1948)](http://www.un.org/Overview/rights.html)  [UN Convention on the Rights of the Child (1989)](http://www.unicef.org/crc/)  [EU Clinical Trials Directive](http://www.eortc.be/Services/Doc/clinical-EU-directive-04-April-01.pdf) (2001/20/EC) |
| Research ethics committees (RECs) | RECs are the bodies that check compliance with all ethical aspects of a research involving human subjects. This encompasses some difficulties. Criticism is being expressed about the relatively haphazard organization of RECs, the lack of diversity in professionalism or representation, and the inadequate professional competence of RECs members for research in human biomonitoring involving human subjects. The main competence in REC’s lays in dealing with clinical studies. In addition, there is only limited uniformity in concepts and decision-making. These committees have also different legal standing, ranging from advisory to legally binding.  However, they could play a key role in assessing the reconciliation of protection of the individual and the scientific needs of the community. They could also provide clarification on possible exemptions to the requirement of informed consent. They could advocate thorough communication to diverse stakeholders (including translation of research results into policy, social science expert groups). In addition also, a close relationship with national data protection authorities would allow to benefit to accrue to all from each other’s knowledge and competence |
| Data protection framework | At EU level, data protection is regulated by Directive 95/46/EC on protection of individuals with regard to the processing of personal data, the so-called Privacy Directive. This Directive is subject to transposition in all EU Member States, and imposes the practice of informed consent, including the right to know one’s own individual results. Also notification of the study to the national data protection supervisory authority is obligatory.  Unfortunately, the transposition of the Directive has lead to divergent interpretations and ambiguities of the regional and national regulations. This creates lack of un-clarity and obstacles for the researcher and inconsistencies in implementation at regional, national and international level. Also, secondary use of data, which is often of utmost importance in human biomonitoring research, may be very difficult. |

**References**

1. **WHO Regional Office for Europe. Health 2020: a European policy framework supporting action across government and society for health and well-being. Copenhagen: WHO Regional Office for Europe** [*http://wwweurowhoint/__data/assets/pdf_file/0006/199536/Health2*](http://wwweurowhoint/__data/assets/pdf_file/0006/199536/Health2) 2013.

2. Bloom D, Cafiero E, Jané-Llopis E, Abrahams-Gessel S, Bloom L, Fathima S, Feigl A, Gaziano T, Mowafi M, Pandya A, et al: **The Global Economic Burden of Noncommunicable Diseases.** *Geneva: World Economic Forum* 2011.

3. Balbus JM, Barouki R, Birnbaum LS, Etzel RA, Gluckman PD, Sr., Grandjean P, Hancock C, Hanson MA, Heindel JJ, Hoffman K, et al: **Early-life prevention of non-communicable diseases.** *Lancet* 2013, **381:**3-4.

4. **Environment and Human Health. Joint EEA-JRC report. EEA Report No 5/2013. Copenhagen: European Environment Agency.** 2013.

5. Norman RE, Carpenter DO, Scott J, Brune MN, Sly PD: **Environmental exposures: an underrecognized contribution to noncommunicable diseases.** *Rev Environ Health* 2013, **28:**59-65.

6. Vineis P, Stringhini S, Porta M: **The environmental roots of non-communicable diseases (NCDs) and the epigenetic impacts of globalization.** *Environ Res* 2014, **133:**424-430.

7. Prüss-Üstün A, Wolf J, Corvalán C, Bos R, Neira M: **Preventing disease through healthy environments A global assessment of the burden of disease from environmental risks. Geneva: World Health Organization** [*http://appswhoint/iris/bitstream/10665/204585/1/9789241565196_engpdf?ua=1*](http://appswhoint/iris/bitstream/10665/204585/1/9789241565196_engpdf?ua=1) 2016.

8. **WHO Regional Office for Europe. Sixth Ministerial Conference on Environment and Health, Copenhagen.** [*http://wwweurowhoint/en/media-centre/events/events/2017/06/sixth-ministerial-conference-on-environment-and-health/read-more*](http://wwweurowhoint/en/media-centre/events/events/2017/06/sixth-ministerial-conference-on-environment-and-health/read-more) 2017a.

9. **WHO Regional Office for Europe. Sustainable Development Goals, Copenhagen** [*http://wwweurowhoint/en/health-topics/health-policy/sustainable-development-goals-sdgs*](http://wwweurowhoint/en/health-topics/health-policy/sustainable-development-goals-sdgs) 2017b.

10. **Ambient air pollution: a global assessment of exposure and burden of disease.** *Geneva: World Health Organization (*[*http://appswhoint/iris/bitstream/10665/250141/1/9789241511353-engpdf?ua=1*](http://appswhoint/iris/bitstream/10665/250141/1/9789241511353-engpdf?ua=1)*)* 2016a.

11. **The public health impacts of chemicals: knowns and unknowns. .** *Geneva: World Health Organization (*[*http://appswhoint/iris/bitstream/10665/206553/1/WHO_FWC_PHE_EPE_1601_engpdf*](http://appswhoint/iris/bitstream/10665/206553/1/WHO_FWC_PHE_EPE_1601_engpdf)*)* 2016b.

12. **WHO Regional Office for Europe. The Minsk Declaration – The Life-course Approach in the Context of Health 2020. Copenhagen: WHO Regional Office for Europe** [*http://wwweurowhoint/__data/assets/pdf_file/0009/289962/The-Minsk-Declaration-EN-rev1pdf?ua=1*](http://wwweurowhoint/__data/assets/pdf_file/0009/289962/The-Minsk-Declaration-EN-rev1pdf?ua=1) 2015a.

13. **WHO Regional Office for Europe, . Strategic Approach to International Chemicals Management: implementation and priorities in the health sector. Meeting report. Copenhagen: WHO Regional Office for Europe** [*http://wwweurowhoint/__data/assets/pdf_file/0015/303036/SAICM-meeting-report-enpdf?ua=1*](http://wwweurowhoint/__data/assets/pdf_file/0015/303036/SAICM-meeting-report-enpdf?ua=1) 2015b.

14. **WHO Regional Office for Europe. Chemical policies and programmes to protect human health and environment in a sustainability perspective. Meeting report, Copenhagen. .** [*http://wwweurowhoint/__data/assets/pdf_file/0009/334665/Chemical-safety-meeting-report_new-coverpdf*](http://wwweurowhoint/__data/assets/pdf_file/0009/334665/Chemical-safety-meeting-report_new-coverpdf)

2017c.

15. **WHO Regional Office for Europe. Implementation of the Minamata Convention in the health sector: challenges and opportunities, Copenhagen. .** [*http://wwweurowhoint/en/health-topics/environment-and-health/health-impact-assessment/publications/2017/implementation-of-the-minamata-convention-in-the-health-sector-challenges-and-opportunities-2017*](http://wwweurowhoint/en/health-topics/environment-and-health/health-impact-assessment/publications/2017/implementation-of-the-minamata-convention-in-the-health-sector-challenges-and-opportunities-2017)

2017d.

16. **OECD Guideline for the testing of chemicals. Combined Chronic toxicity/carcinogenicity studies. TG 453. .** 2009.

17. Teitelbaum SL, Li Q, Lambertini L, Belpoggi F, Manservisi F, Falcioni L, Bua L, Silva MJ, Ye X, Calafat AM, Chen J: **Paired Serum and Urine Concentrations of Biomarkers of Diethyl Phthalate, Methyl Paraben, and Triclosan in Rats.** *Environmental health perspectives* 2016, **124:**39-45.

18. Dong H, Lei J, Ding L, Wen Y, Ju H, Zhang X: **MicroRNA: function, detection, and bioanalysis.** *Chem Rev* 2013, **113:**6207-6233.

19. Reid G, Kirschner MB, van Zandwijk N: **Circulating microRNAs: Association with disease and potential use as biomarkers.** *Crit Rev Oncol Hematol* 2011, **80:**193-208.

20. Pant S, Hilton H, Burczynski ME: **The multifaceted exosome: biogenesis, role in normal and aberrant cellular function, and frontiers for pharmacological and biomarker opportunities.** *Biochem Pharmacol* 2012, **83:**1484-1494.

21. Thomas P, Holland N, Bolognesi C, Kirsch-Volders M, Bonassi S, Zeiger E, Knasmueller S, Fenech M: **Buccal micronucleus cytome assay.** *Nat Protoc* 2009, **4:**825-837.

22. Bolognesi C, Knasmueller S, Nersesyan A, Thomas P, Fenech M: **The HUMNxl scoring criteria for different cell types and nuclear anomalies in the buccal micronucleus cytome assay - an update and expanded photogallery.** *Mutat Res* 2013, **753:**100-113.

23. Bolognesi C, Roggieri P, Ropolo M, Thomas P, Hor M, Fenech M, Nersesyan A, Knasmueller S: **Buccal micronucleus cytome assay: results of an intra- and inter-laboratory scoring comparison.** *Mutagenesis* 2015, **30:**545-555.

24. Bolognesi C, Knasmueller S, Nersesyan A, Roggieri P, Ceppi M, Bruzzone M, Blaszczyk E, Mielzynska-Svach D, Milic M, Bonassi S, et al: **Inter-laboratory consistency and variability in the buccal micronucleus cytome assay depends on biomarker scored and laboratory experience: results from the HUMNxl international inter-laboratory scoring exercise.** *Mutagenesis* 2016.

25. Holland N, Bolognesi C, Kirsch-Volders M, Bonassi S, Zeiger E, Knasmueller S, Fenech M: **The micronucleus assay in human buccal cells as a tool for biomonitoring DNA damage: the HUMN project perspective on current status and knowledge gaps.** *Mutat Res* 2008, **659:**93-108.

26. Chassang G: **The impact of the EU general data protection regulation on scientific research** *ecancermedicalscience* 2017, **11:**709.
